# Effects of Chemicals in Reporter Gene Bioassays with Different Metabolic Activities Compared to Baseline Toxicity

**DOI:** 10.1021/acs.chemrestox.4c00017

**Published:** 2024-04-23

**Authors:** Julia Huchthausen, Jenny Braasch, Beate I. Escher, Maria König, Luise Henneberger

**Affiliations:** † Department of Cell Toxicology, Helmholtz Centre for Environmental Research − UFZ, Permoserstr. 15, 04318 Leipzig, Germany; ‡ Environmental Toxicology, Department of Geosciences, Eberhard Karls University Tübingen, 72076 Tübingen, Germany

## Abstract

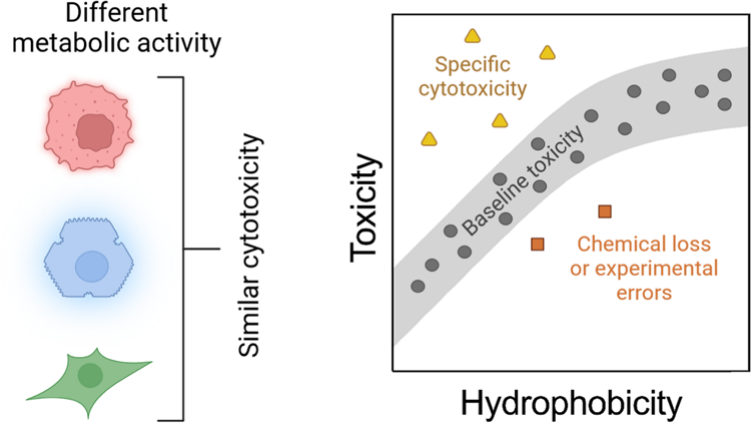

High-throughput cell-based bioassays are used for chemical screening and risk assessment. Chemical transformation processes caused by abiotic degradation or metabolization can reduce the chemical concentration or, in some cases, lead to the formation of more toxic transformation products. Unaccounted loss processes may falsify the bioassay results. Capturing the formation and effects of transformation products is important for relating the *in vitro* effects to *in vivo.* Reporter gene cell lines are believed to have low metabolic activity, but inducibility of cytochrome P450 (CYP) enzymes has been reported. Baseline toxicity is the minimal toxicity a chemical can have and is caused by the incorporation of the chemical into cell membranes. In the present study, we improved an existing baseline toxicity model based on a newly defined critical membrane burden derived from freely dissolved effect concentrations, which are directly related to the membrane concentration. Experimental effect concentrations of 94 chemicals in three bioassays (AREc32, ARE-*bla* and GR-*bla*) were compared with baseline toxicity by calculating the toxic ratio (TR). CYP activities of all cell lines were determined by using fluorescence-based assays. Only ARE-*bla* showed a low basal CYP activity and inducibility and AREc32 showed a low inducibility. Overall cytotoxicity was similar in all three assays despite the different metabolic activities indicating that chemical metabolism is not relevant for the cytotoxicity of the tested chemicals in these assays. Up to 28 chemicals showed specific cytotoxicity with TR > 10 in the bioassays, but baseline toxicity could explain the effects of the majority of the remaining chemicals. Seven chemicals showed TR < 0.1 indicating inaccurate physicochemical properties or experimental artifacts like chemical precipitation, volatilization, degradation, or other loss processes during the *in vitro* bioassay. The new baseline model can be used not only to identify specific cytotoxicity mechanisms but also to identify potential problems in the experimental performance or evaluation of the bioassay and thus improve the quality of the bioassay data.

## Introduction

Risk assessment of chemicals using high-throughput tests is becoming more and more important since the number of chemicals is increasing year by year. The conventional approach of using animal studies to assess the risk of chemicals to humans has been used for many years. However, a comprehensive risk assessment of chemicals using animal-based studies is not feasible, as animal testing for the risk assessment of single chemicals is extremely costly and can take several years to complete.^1^

Therefore, there has been a recent shift in the focus of risk assessment to so-called “New Approach Methodologies” (NAMs) which include a wide range of experimental approaches such as *in vitro* bioassays and omics methods, as well as *in silico* approaches such as quantitative structure–activity relationships (QSARs), and machine learning approaches. While these methods are not new, a combination of *in silico*, *in chemico* and *in vitro* approaches can provide information to assess the risks of even data-poor chemicals and reduce or eliminate the use of animals.^2−4^ The PrecisionTox initiative started in February 2021 and was funded by the European Commission as part of the Horizon 2020 program for the development of NAMs for testing the safety of chemicals. The goal of PrecisionTox is to understand the systemic toxicity of chemicals by applying evolutionary principles to compare toxicological responses in different species. The concept of “toxicity by descent” suggests that the mechanisms of chemical toxicity may be similar in distantly related species due to a shared biology. PrecisionTox uses five model species (*Drosophila melanogaster*, *Caenorhabditis elegans*, *Daphnia magna*, and embryos of *Xenopus laevis* and *Danio rerio*) as well as human cell lines for high-throughput testing. The project integrates phenotype, metabolome, and transcriptome analysis to identify toxicity pathways.^5^ The present study investigated the *in vitro* effects of 94 chemicals with diverse physicochemical properties selected within the PrecisonTox project for analysis in three different *in vitro* cell-based bioassays.

A major impediment for the use of *in vitro* bioassays for risk assessment is the lack of exposure assessment of the chemicals. The prerequisite to generate reliable *in vitro* data is a stable chemical concentration over the course of the assay. Chemicals are subject to a variety of loss processes during the assay, which have been discussed in numerous publications. Binding to medium components,^6^ binding to the plate material,^7^ volatilization,^8^ and abiotic^9^ and biotic^10^ degradation processes can only be reliably excluded for the large spectrum of chemicals by measuring the chemical concentration. Although methods exist for the experimental measurement of chemical concentration in 96- and 384-well plates,^11,12^ these methods are very time-consuming and labor-intensive and therefore are not compatible with high-throughput screening of hundreds of chemicals. Mass-balance models can confidently predict loss processes due to partitioning/binding to plates, medium, and loss to air.^13,14^

An indirect way to estimate if there might be problems associated with loss of chemicals is to compare the measured *in vitro* cytotoxicity data with predicted baseline toxicity,^8^*i.e*., the minimum toxicity of every chemical.^15^ Baseline toxicity is caused by the incorporation of the chemical into the cell membrane and its resulting destabilization. It occurs at critical membrane burdens (CMBs) that are constant and independent of the chemical properties. Linear low concentration–response curves have been established for cell-based reporter gene bioassays to avoid artifacts caused by the so-called cytotoxicity burst.^16^ Furthermore, problems with solubility can be avoided by focusing on the low concentration range. Therefore, CMBs for reporter gene cell lines are determined at the 10% inhibitory concentration of cell viability (IC_10_). Escher et al.^8^ estimated a CMB (IC_10,membrane_) of 69 mmol/L_lip_ for cell-based bioassays, which appeared to be not only independent of the chemicals but also of the cell types.

Lee et al.^17^ developed an empirical QSAR model for the prediction of baseline toxicity in reporter gene cell lines, which only requires the liposome–water distribution ratio (*D*_lip/w_) of the chemical as an input parameter. The toxic ratio (TR) can be calculated to compare the measured toxicity with baseline toxicity. If chemicals show higher toxicity than baseline toxicity (TR > 10), this is indicating a specific cytotoxicity mechanism.^18^ If chemicals show lower toxicity than baseline toxicity (TR < 0.1), this may be an artifact caused by a loss of the chemical in the course of the bioassay. However, the existing empirical QSAR model is limited to chemicals with log *D*_lip/w_ > 0 and has not been validated for the prediction of the toxicity of anions.^17^ Recently, Qin et al. developed a baseline toxicity QSAR for neutral and anionic per- and polyfluorinated substances (PFAS) based on the same critical membrane concentration.^19^

The IC_10,membrane_ of 69 mmol/L_lip_ used in these mass-balance models was derived from nominal effect concentrations (IC_10,nom_). An improved estimation of IC_10,membrane_ is possible using measured freely dissolved effect concentrations (IC_10,free_), as these account for distribution processes of the chemicals between water and medium components. IC_10,membrane_ can be directly derived from IC_10,free_ using the *D*_lip/w_ values of the test chemicals.
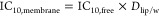
1IC_10,free_ equals IC_10,nom_ for hydrophilic chemicals that do not show significant binding to medium components.^11^ This premise allows for the improvement of baseline toxicity predictions by redefining IC_10,membrane_ based on IC_10,free_ of hydrophilic chemicals. In the present study, a subset of nonvolatile, hydrophilic chemicals with measured *D*_lip/w_ and without specific effects was selected and their cytotoxicity was measured in the AREc32, ARE-*bla*, and GR-*bla* assay to develop this refined baseline toxicity model. Additionally, ionizable chemicals with measured IC_10,free_ values were included to generate a QSAR model that is also applicable to anionic chemicals.

Reporter gene cells are often derived from cancer cell lines and assumed to have very limited metabolic activity.^20−23^ However, it has been shown that the formation of cytochrome P450 (CYP) enzymes can be induced by xenobiotic chemicals.^10,24,25^ Metabolization can lead to the loss of the parent chemical. For example, the metabolic transformation of benzo[*a*]pyrene led to a decrease in cellular concentration over time as shown by Fischer et al.^10,26^ meaning that cellular metabolism may lead to false effect concentrations. However, the formation of reactive metabolites with a higher toxicity is also possible. The comparison of effect data from cell lines with different metabolic activity can therefore provide an indication of the relevance of metabolization of the test chemicals in *in vitro* bioassays.

In this study, three representative reporter gene cell lines were selected based on three frequently used cell lines with different origins. AREc32 is based on the MCF-7 breast cancer cell line, ARE-*bla* is based on a HepG2 liver cell line and GR-*bla* is based on a HEK293T embryonic kidney cell line. Over half of the bioassays of the Tox21 test battery^27^ are based on HEK293T (42%) and HepG2 (14%) cells.^10^ Two of the cell lines (AREc32 and ARE-*bla*) carry a reporter gene for the activation of the oxidative stress response^28,29^ and one cell line (GR-*bla*) carries a reporter gene for the glucocorticoid receptor,^30^ representing two important toxicological end points. The three selected cell lines showed different activity and inducibility of CYP1 in a previous study.^10^

Cellular CYP activities without and with chemical induction were investigated with fluorescence-based assays. 7-Ethoxy-resorufin-*O*-deethylase (EROD) assay was used to measure CYP1A1/2 activity,^31^ 7-ethoxy-4-trifluoromethylcoumarin-*O*-deethylase (EFCOD) assay was used to measure CYP2B6 activity,^32^ and 7-benzyloxy-4-trifluoromethylcoumarin-*O*-debenzyloxylase (BFCOD) assay was used to measure CYP3A4/5 activity.^33^

The objectives of this study were: (1) to develop a novel baseline toxicity model for *in vitro* bioassays that is applicable to hydrophilic and charged chemicals; (2) to determine the metabolic activity of three reporter gene cell lines with different cellular origins; (3) to measure the effects of 94 chemicals with diverse physicochemical properties in high-throughput screening in the three cell lines; and (4) to compare the cytotoxicity measured in the three cell lines with each other and with baseline toxicity predictions. Thus, it may be possible to correlate the *in vitro* effects with the metabolic activity of the cell lines if the resulting metabolites lead to higher or lower effects. This combined approach can increase confidence in *in vitro* data, as valuable information on chemical exposure can be obtained by careful analysis of in *vitro* effect concentrations. Possible loss processes that would otherwise have remained unobserved can be uncovered, thus, preventing misinterpretation of *in vitro* data.

## Material and Methods

### Chemicals

A total of 94 chemicals (Table 1) were tested in three bioassays. Volatile or very hydrophobic chemicals were not included. More information on the test chemicals can be found in the Supporting Information in Table S1. The method for p*K*_a_ measurement is described in the literature^12,34^ and the method for log *K*_ow_ measurement can be found in the Supporting Information (Text S1). Chemicals were either dosed as stock solutions in dimethyl sulfoxide (DMSO), methanol, or water or directly dissolved in bioassay medium depending on the dosing concentration and chemical solubility. Information on the additional hydrophilic chemicals (log *K*_lip/w_ between −1.04 and 0.81) for the development of a baseline QSAR model can be found in Table S2. Chemicals for CYP activity assays were purchased from Sigma-Aldrich (omeprazole, benzo[*a*]pyrene, resorufin, 7-ethoxyresorufin, 7-hydroxy-4-trifluoromethylcoumarin) with a purity ≥95% and from Chemodex (7-ethoxy-4-trifluoromethylcoumarin, 7-benzyloxy-4-trifluoromethylcoumarin) with a purity ≥98%. Rat liver S9 was purchased from Molecular Toxicology and nicotinamide adenine dinucleotide phosphate tetrasodium salt (NADPH, purity ≥95%) was obtained from Roth.

**Table 1:** Chemicals of This Study

chemical	ID	chemical	ID
1,2-dimethyl-1*H*-imidazole	12DMIZ	genistein	GEN
1-ethyl-1*H*-imidazole	1E1HIZ	haloperidol	HPD
1*H*-imidazole-1-propanamine	1HIZ1P	HC yellow 13	HCY
1-methylimidazole	1MIZ	hexachlorophene	HCP
1-vinylimidazole	1VIZ	hydroxyurea	HU
2-ethyl-4-methyl-1*H*-imidazole	2E4MIZ	imazalil	IMZ
2-ethylimidazole	2EIZ	imidacloprid	IMI
2-methylimidazole	2MIZ	imidazole	IZ
4-methylimidazole	4MIZ	lidocaine	LIDO
5,5-diphenylhydantoin	55DH	mebendazole	MBZ
5-fluorouracil	5FU	methacrylamide	MAA
acetaminophen	APAP	methimazole	MMI
acrylamide	AA	methotrexate	MTX
all-trans retinoic acid	ATRA	*N*-(butoxymethyl)acrylamide	NBuAA
arsenic(III) oxide	As_2_O_3_	*N*-(isobutoxymethyl)acrylamide	NIAA
aspartame	ASP	*N,N*′-bis(2-hydroxyethyl)-2-nitro-p-phenylenediamine	NBNP
atorvastatin	ATO	*N,N*-diethylacrylamide	NDAA
atrazine	ATZ	*N,N*-dimethylacetamide	DMA
azacytidine	AZA	*N,N*-dimethylformamide	DMF
azoxystrobin	AZ	*N,N*′-methylenebisacrylamide	NMBAA
bisphenol A	BPA	nicotine	NIC
bromodeoxyuridine	BDU	niflumic acid	NIFA
butoxyethanol	BE	*N*-methylaniline	NMA
cadmium chloride	CdCl_2_	*N*-methylolacrylamide	NMAA
caffeine	CAF	*o*-aminophenol	oAP
camptothecin	CPT	PCB28	PCB28
carbamazepine	CBZ	perfluorooctanoic acid	PFOA
carbendazim	CBD	picoxystrobin	PXS
chlorpromazine	CPZ	pirinixic acid	WY-14643
chlorpyrifos	CP	pregnenolone	PREG
chlorpyrifos-oxon	CPO	propofol	PPF
citalopram	CT	propylthiouracil	PTU
clofibric acid	CFA	rotenone	RTN
colchicine	CC	sodium arsenite	NaAsO_2_
cyclophosphamide	CPA	tamoxifen	TAM
cyclosporin A	CSA	tebuconazole	TCZ
cyproconazole	CPCZ	tetracycline	TET
cytosinearabinoside	CARA	tetraethylthiuram disulfide	TETD
dexamethasone	DEXA	thiamethoxam	TMX
diclofenac	DCL	tigecycline	TG
dimethyl sulfoxide	DMSO	toluene-2,5-diamine	T25D
diphenylamine	DPA	triadimenol	TDM
ethoprophos	EPP	tributyltin	TBT
ethylenethiourea	ETU	trichlorfon	TCF
fingolimod	FGM	triethyl-tin bromide	TEtT
fipronil	FIP	valproic acid	VPA
fluoxetine	FLX	verapamil	VRP

### Materials

All components of the bioassay media as well as CellSensor ARE-*bla* and GeneBLAzer GR-UAS-*bla* cells were purchased from Thermo Fisher Scientific. AREc32 cells were purchased from Cancer Research UK. Poly-d-lysine-treated black 384-well plates with a clear bottom (Product No. 356663) for ARE-*bla* and GR-*bla* assay and white 384-well plates with a clear bottom (Product No. 3765) for AREc32 were purchased from Corning. Water was obtained from a Milli-Q water purification system from Merck.

### *In Vitro* Bioassays

All chemicals were tested in the ARE-*bla* and AREc32 assay for the detection of the oxidative stress response activation *via* the Nuclear Factor Erythroid 2 related Factor 2/Kelch-like ECH-associated protein 1 (Nrf-2/Keap-1) pathway. GR-*bla* cells carry a reporter gene for the glucocorticoid receptor (GR). The procedure of the *in vitro* bioassays has been described in detail in the literature.^35−37^ ARE-*bla* bioassay medium (90% DMEM phenol red-free, 10% dialyzed fetal bovine serum (d-FBS), 0.1 mM nonessential amino acids, 25 mM HEPES, 1 mM sodium pyruvate, 4.97 mM GlutaMAX, 100 U/mL penicillin-streptomycin), GR-*bla* bioassay medium (98% Opti-MEM, 2% charcoal-stripped FBS (cs-FBS), 100 U/mL penicillin-streptomycin), and AREc32 bioassay medium (90% DMEM with GlutaMAX, 10% FBS, 100 U/mL penicillin-streptomycin) were prepared. A MultiFlo dispenser (Biotek, Vermont) was used to dispense 30 μL of a cell suspension per well. Final cell counts were 4100 cells/well (ARE-*bla*), 6000 cells/well (GR-*bla*), and 2650 cells/well (AREc32). Plates were incubated for 24 h at 37 °C and 5% CO_2_, and cell confluency was measured before and 24 h after chemical dosing using an IncuCyte S3 Live-Cell Analysis System (Essen BioScience, Sartorius) in HD phase contrast mode with 10× magnification at room temperature. Cell confluency was determined from customized confluency masks created with the basic analyzer software (Incucyte 2023A). The chemicals were either directly dissolved in bioassay medium or dosed as DMSO, methanol or water stock solutions. The final DMSO content in the wells was kept below 0.5%, the methanol content was kept below 1%. For acids and bases which are charged at pH 7.4, an equimolar aliquot of sodium hydroxide or hydrochloric acid was added to the dosing vials. Dosing plates containing the chemicals in serial dilution were prepared using a Hamilton Microlab Star robotic system (Hamilton, Bonaduz, Switzerland). *tert*-Butylhydroquinone (final concentration in cell plate 1.73 × 10^–5^ M – 1.35 × 10^–8^ M) was used as positive control for ARE-*bla* and AREc32 and dexamethasone (final concentration in cell plate 5.05 × 10^–8^ M – 1.53 × 10^–12^ M) was used as positive control for GR-*bla*. The diluted chemicals were dispensed in duplicate by transferring 10 μL from the dosing plates to the cell plate twice. Chemicals with high air–water partitioning constants (*K*_aw_) (NMA, NIC, IMZ and PCB28) were tested on a separate plate, which was sealed with a gas-permeable plate cover (Product No. 4ti-0516/384, Azenta Life Sciences), with one row of bioassay medium between the rows containing the chemicals. The cell plates were incubated at 37 °C and 5% CO_2_ for 24 h after dosing. Cytotoxicity was determined by comparing the confluency of the dosed wells with control wells without chemical addition. Reporter gene activation was quantified as described in the literature.^35−37^ Briefly, for AREc32 the medium was removed, and the cells were washed 3 times with phosphate-buffered saline (PBS, 137 mM NaCl, 2.7 mM KCl, 8.1 mM disodium phosphate dihydrate, 1.8 mM potassium dihydrogen phosphate). 10 μL of lysis buffer (50 mM Tris, 2% Triton-X 100, 4 mM ethylenediaminetetraacetic acid (EDTA), 4 mM DL-dithiothreitol (DTT), 20% glycerol) were added to each well and incubated at room temperature and 1500 rpm (BioShake iQ, QInstruments) for 20 min. 40 μL of d-luciferin buffer (pH 7.7–7.8, 20 mM tricine, 2.67 mM magnesium sulfate pentahydrate, 33.3 mM DTT, 0.1 mM EDTA, 0.261 mM coenzyme A, 0.53 mM adenosine 5′-triphosphate, 0.235 mM d-luciferin added immediately before use) were added to all wells. The plates were shaken for 30 s and 1000 rpm (BioShake iQ, QInstruments), and luminescence was detected with a multimode plate reader (Tecan Reader Infinite 1000 Pro). For ARE-*bla* and GR-*bla*, ToxBLAzer working solution (Product No. K1138, Thermo Fischer) was prepared according to the manufacturer’s protocol. 8 μL of working solution were added per well and fluorescence was measured (Ex/Em: 409/460; 409/530 and 590/665 nm). Plates were incubated for 2 h at room temperature in the dark, and fluorescence was measured again. ToxBLazer reagent contains also a cytotoxicity indicator that detects cell membrane integrity and esterase activity as a measure of cytotoxicity next to reporter gene activation. All chemicals were tested in all assays in three independent replicates.

### Baseline Toxicity

The target site of baseline toxicants is the membrane; therefore, baseline toxicity can be predicted if the CMB is known. Since baseline toxicity is unspecific, the CMB should be similar for all chemicals and cell lines.^8,38^ Partitioning processes of the chemicals to proteins or lipids of the bioassay medium can reduce the freely dissolved effect concentration for cytotoxicity (IC_10,free_) of the chemicals and must be considered for the determination of the CMB. Ideally, measured IC_10,free_ values should be used to calculate the CMB, as these represent the actual available concentration that can partition into the cell membrane. The CMB can also be determined from neutral, hydrophilic chemicals, as these do not bind to medium components or only to a negligible extent, and therefore the nominal effect concentration IC_10,nom_ equals IC_10,free_ and internal and external freely dissolved concentrations can be assumed to be equal.

Fourteen chemicals with measured log *K*_lip/w_ values between −1.04 and 0.81 were selected to calculate the IC_10,membrane_ (Table S2). All chemicals were tested once in serial and twice in linear dilution in the AREc32, ARE-*bla* and GR-*bla* assay. The assays were performed as described above, but only cytotoxicity was measured *via* confluency measurement, not reporter gene activation or ToxBLAzer cytotoxicity. Additionally, 15 chemicals with experimental log *D*_lip/w_ (0.08–4.73), previously measured IC_10_,_free_ and without known specific cytotoxicity were included for the calculation of IC_10,membrane_.^39^

For more hydrophobic chemicals, the nominal concentration of the test chemicals causing baseline toxicity (IC_10,nom,baseline_) can be calculated from the IC_10,membrane_ using a mass-balance model to estimate chemical partitioning to medium components.^13^ Partitioning to cells is negligible in protein-rich bioassay media as they only constitute a very small percentage of the total protein and lipid volume of the bioassay.^19^
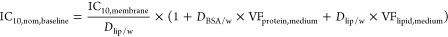
2As there is a linear relationship between the distribution ratios of the test chemicals to bovine serum albumin (*D*_BSA/w_) and the distribution ratios to liposomes (*D*_lip/w_), the only input parameters for the mass-balance model are the volume fractions (VF) of protein and lipid of the media used which were obtained from the literature^19^ (Table S3) and the *D*_lip/w_ of the test chemicals. Experimental *D*_lip/w_ were obtained from the literature or predicted using Linear Solvation Energy Relationship (LSER) or QSAR models and are summarized in Table S1.^40,41^

A linear QSAR for the calculation of log *D*_BSA/w_ from log *D*_lip/w_ has been developed for neutral chemicals (eq 3).^42^
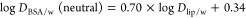
3Recently, a similar QSAR has been developed for anionic perfluoroalkyl and polyfluoroalkyl substances (PFAS). However, it will be evaluated if this QSAR is also applicable to other organic anions tested in the present study (eq 4).^19^
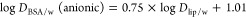
4

By insertion of eqs 3 or 4 in eq 2, a nominal baseline toxicity QSAR can be derived for neutral chemicals (eq 5) and anionic chemicals (eq 6) which requires only *D*_lip/w_ as input parameter.
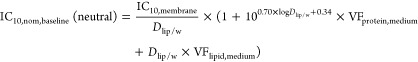
5
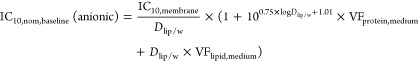
6

The toxic ratio (TR) was calculated to compare the measured cytotoxicity (IC_10_) with the baseline toxicity (IC_10,baseline_). A TR between 0.1 and 10 means that the chemical is a baseline toxicant, and a TR higher than 10 indicates a specific mode of action.
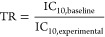
7

### Metabolic Characterization

To determine cytochrome P450 (CYP) enzyme activity and inducibility of CYP activity, the fluorescence-based EROD, EFCOD and BFCOD assays were used. The substrates are metabolized by different CYP enzymes (CYP1A1/2, CYP2B6 and CYP3A4/5) and form fluorescent metabolites. The fluorescence of the respective metabolites can be detected, and thus the CYP activity can be quantified.

For all three assays, 90 μL of cell suspension were dispensed in black 96-well plates (Corning, AREc32 Product No. 3603, ARE-*bla* and GR-*bla* Product No. 354640) with 11000 cells/well (AREc32 and ARE-*bla*) or 20000 cells/per well (GR-*bla*). The plates were incubated at 37 °C and 5% CO_2_. After 24 h, 30 μL of a dilution of the CYP inducers omeprazole or benzo[*a*]pyrene in bioassay medium was dosed to the cells. The concentration in the plate was below cytotoxicity (IC_10_) for all three cell lines (Text S2 and Figures S1–S3). The final chemical concentrations can be found in Table S4. Wells with only the medium were included to determine basal CYP activity. After chemical dosing, the plates were incubated at 37 °C and 5% CO_2_ for 24 h. The confluency of the cells was measured before and after incubation using an IncuCyte S3 Live-Cell Analysis System (Essen BioScience, Sartorius) to monitor the cell viability and calculate the cell growth. For the detection of CYP activity, the medium was removed from all wells and the cells were washed with 120 μL of PBS twice. 7-Ethoxyresorufin (ETX), 7-ethoxy-4-trifluoromethylcoumarin (EFC) and 7-benzyloxy-4-trifluoromethylcoumarin (BFC) working solutions in PBS were prepared. The concentrations of ETX, EFC and BFC were optimized (Text S2 and Figure S4) and were 2 μM (ETX) and 5 μM (EFC and BFC). Calibration standards of resorufin and 7-hydroxy-4-trifluoromethylcoumarin (HFC) were prepared by serial dilution in PBS. The concentration range of the calibration standards was 3.13 × 10^–7^ M – 2.45 × 10^–10^ M (resorufin) and 1.00 × 10^–6^ M – 7.81 × 10^–11^ M (HFC). 120 μL of either EROD, EFCOD and BFCOD working solution were added to all wells containing cells and to all control wells without cells. 120 μL of each calibration standard were added to empty wells. The fluorescence intensity of resorufin (Ex/Em: 560:584 nm) or HFC (Ex/Em: 405:520 nm) was measured in a preheated microplate reader (plate reader Infinite M1000 PRO, Tecan) at 37 °C for 10 min with measurement intervals of 30 s. The formed resorufin or HFC amount (*n*_resorufin_ or *n*_*H*FC_) was calculated after background subtraction from the control wells without cells with the respective calibration curve. From the slope (*k*) of a linear regression of *n*_resorufin_ or *n*_HFC_ against time (*t*) with an intercept of *n*_resorufin_ or *n*_HFC_ at the beginning of measurement (*n*_resorufin_ or *n*_HFC_)_t0_ (eq 8), the CYP activity was determined as the amount of resorufin or HFC formed per minute (*n*_resorufin_ or *n*_HFC_ mol min^–1^)*_t_*.
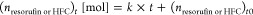
8This was normalized to the mass of protein in the wells (mg_protein_) which was calculated from the cell number after 48 h and the protein content of the cells which were 0.70 mg_protein_ per 10^6^ cells for AREc32,^6^ 0.21 mg_protein_ per 10^6^ cells for ARE-*bla*, and 0.45 mg_protein_ per 10^6^ cells for GR-*bla*.^10^

As positive control, EROD, EFCOD and BFCOD assay were performed with rat liver S9 (Moltox, protein content 39.3 mg/mL). Rat liver S9 was diluted with PBS to a final protein concentration of 0.1 mg/mL. NADPH was added at a final concentration of 80 μM. 60 μL of the mix was added to the wells of a black 96-well plate (Corning, Product No. 3603). EROD, EFCOD and BFCOD working solutions and resorufin and HFC calibration standards were prepared as described above. 60 μL of either EROD, EFCOD and BFCOD working solution was added to all wells containing S9 and to all control wells, and 120 μL of the respective calibration standards was added to empty wells. CYP activity was measured as described above.

### Data Evaluation

Bioassay raw data was processed using an automatic KNIME (version 4.6.1) workflow, and GraphPad Prism (version 10.1.0) was used for data visualization. Cytotoxicity and reporter gene activation were plotted against the chemical concentration to derive concentration–response curves for all chemicals.

The concentration at which cell viability was reduced by 10% (IC_10_) was calculated from the slope of the regression of the linear range of the concentration–response curves using eq 9.^43^
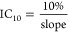
9The induction ratio (IR) was calculated for the evaluation of the activation of the oxidative stress response, and the EC_IR1.5_ (eq 10) was derived from the slope of the linear part of concentration–response curve. Only concentrations below 10% cytotoxicity were used for the calculation.^37^
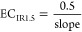
10For activation of GR, the concentration at which 10% of the maximum effect was reached (EC_10_) was calculated from the slope of the linear part of the concentration–response curve. Only concentrations below 10% of cytotoxicity were used for the calculation. The maximum effect was calculated from the concentration–response curve of the reference chemical dexamethasone.
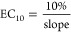
11The specificity ratio (SR) was used to compare the reporter gene activation with cytotoxicity (SR_cytotoxicity_) or baseline toxicity (SR_baseline_).^16^ An SR > 10 means that the reporter gene activation is specific and not linked to cytotoxicity.
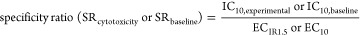
12

## Results and Discussion

### Baseline Toxicity

Concentration–response curves (CRCs) of 14 hydrophilic chemicals in all three assays are shown in Figures S5 and S7, and IC_10_ values are listed in Table S5. Measured IC_10,free_ of 15 additional chemicals from Huchthausen et al.^39^ are shown in Table S6.

**Figure 1:**
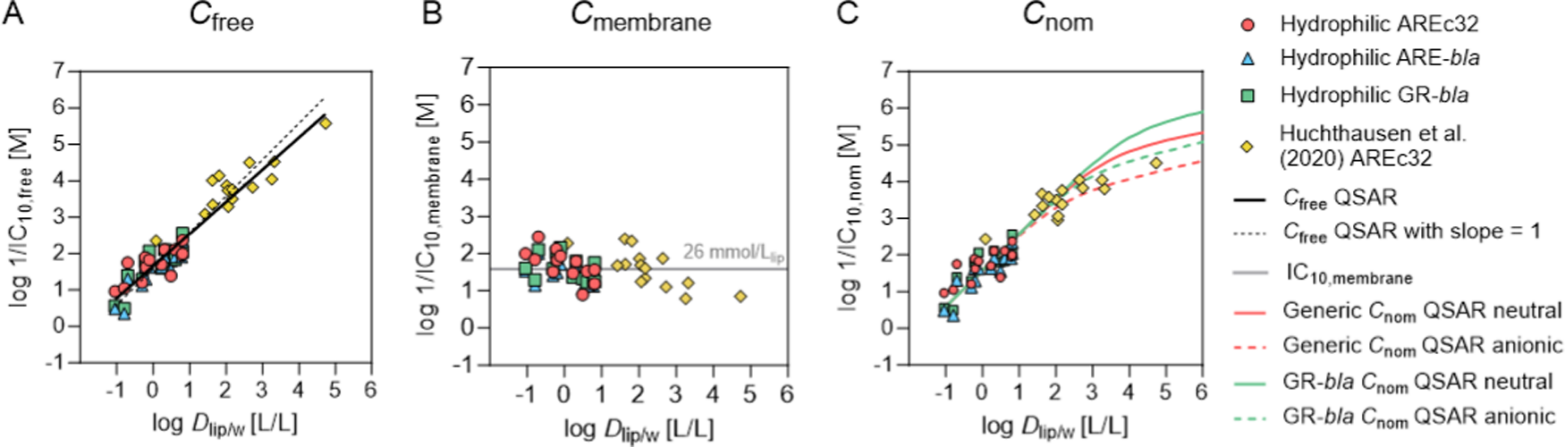
Experimental derivation of nominal baseline toxicity QSARs for neutral and anionic chemicals for AREc32, ARE-*bla*, and GR-*bla* cell lines. (A) Logarithmic reciprocal IC_10,free_ (log 1/IC_10,free_) of test chemicals were plotted against logarithmic liposome–water distribution ratios (log *D*_lip/w_). The black solid line is the linear regression of the data points, and the dotted black line is the linear regression with a slope fixed to 1, which was used to derive the critical membrane concentration for baseline toxicity (IC_10,membrane_). (B) Logarithmic reciprocal IC_10,membrane_ (log 1/IC_10,membrane_) of test chemicals were plotted against log *D*_lip/w_. The solid gray line indicates the constant CMB of 26 mmol/L_lip_ derived from the linear regression from A. (C) Logarithmic reciprocal IC_10,nom_ (log 1/IC_10_) of test chemicals plotted against log *D*_lip/w_ derived with the mass-balance models for neutral and anionic chemicals. The red solid line indicates the generic QSAR for AREc32 and ARE-bla for neutral chemicals, and the red dotted line indicates the generic anionic QSAR. The green solid line indicates the QSAR for GR-*bla* for neutral chemicals, and the green dotted line indicates the anionic QSAR for GR-*bla*.

Logarithmic reciprocal IC_10,free_ of all chemicals were plotted against logarithmic *D*_lip/w_ and linear regression of all data points had a slope of 0.8837 ± 0.0403 and an intercept of 1.658 ± 0.0550 (Figure 1A). As the CMB is independent of hydrophobicity, the ideal slope of the regression is 1. The measured slope is close to the ideal slope, and the deviation is probably caused by variation of the cytotoxicity or concentration measurements. However, it was in line with QSARS for other species,^44,45^ so the slope of the regression was set to 1 resulting in an intercept of 1.585 ± 0.0519 which converts to a constant IC_10,membrane_ of 26 ± 3.3 mmol/L_lip_ (Figure 1B). This CMB is 2.6 times lower than the previously reported CMB of 69 mmol/L_lip_. The earlier IC_10,membrane_ was only modeled from IC_10,nom_ of a set of chemicals with a limited hydrophobicity range. Therefore, the new IC_10,membrane_ of 26 mmol/L_lip_ can be considered more reliable and robust, but should be validated with measured *C*_free_ for more hydrophobic chemicals in future studies.

The newly derived IC_10,membrane_ as well as the respective protein and lipid contents of the media (Table S3) were inserted in eqs 5 and 6 resulting in the new nominal baseline QSAR equations for neutral (eqs 13 and 15) and anionic chemicals (eqs 14 and 16).
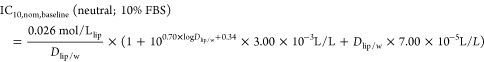
13
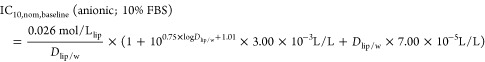
14
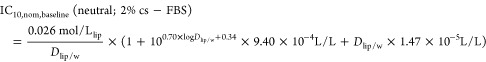
15
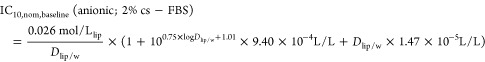
16Logarithmic reciprocal nominal IC_10_ values of the test chemicals were plotted against *D*_lip/w_ (Figure 1C). AREc32 and ARE-*bla* assay media had a similar protein and lipid content as both were supplemented with 10% FBS or d-FBS (Table S3), so a generic QSAR was developed for both assays for neutral (eq 13) and anionic (eq 14) chemicals (Figure 1C, red lines). GR-*bla* medium had a lower protein and lipid content as it was supplemented with 2% cs-FBS (Table S3), so a separate GR-*bla* QSAR was developed for neutral (eq 15) and anionic (eq 16) chemicals (Figure 1C, green lines).

### Metabolic Characterization

**Figure 2:**
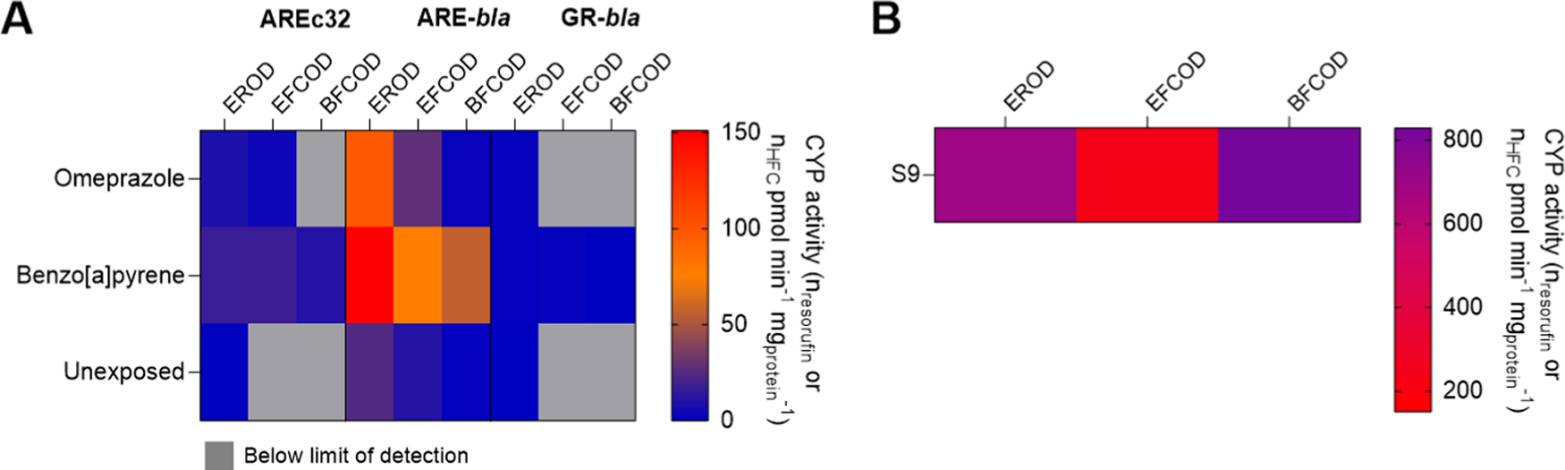
Results of EROD, EFCOD and BFCOD assay for AREc32, ARE-*bla*, and GR-*bla* cells without chemical exposure and after exposure to omeprazole or benzo[*a*]pyrene (A) and for rat liver S9 as a positive control (B). CYP activity was measured as the amount of resorufin (*n*_resorufin_, EROD) or the amount of 7-hydroxy-4-trifluoromethylcoumarin (*n*_HFC_, EFCOD and BFCOD) formed per minute and per mg_protein_.

Reporter gene cell lines are often cited to have a lack of metabolic capacity.^20,22,23^ However, it has also been reported that the activity of CYP enzymes in cell lines can be activated by chemicals.^10,24,25^ Different metabolic activity makes it difficult to compare *in vitro* data from different cell lines because metabolization of the test chemicals affects *in vitro* effect data. Fischer et al. have shown that the medium and cellular concentration of benzo[*a*]pyrene decreased and the concentration of formed metabolites increased significantly in the course of an *in vitro* bioassay with AREc32 and ARE-*bla* cells.^10^ Therefore, in this study, CYP enzyme activity of all three cell lines was measured using three different assays (EROD, EFCOD, and BFCOD assays). Basal CYP activities were low for all cell lines and were below the detection limit for EFCOD and BFCOD assay in case of AREc32 and GR-*bla*. ARE-*bla* had the highest basal EROD activity with 24.36 pmol resorufin formed per minute per mg_protein_ (Figure 2A). CYP activities were also measured after incubation with the CYP inducers benzo[*a*]pyrene (B[*a*]P)^10^ and omeprazole.^24^ B[*a*]P induces CYP1 gene expression *via* binding to the aryl hydrocarbon receptor (AhR)^25,46^ while omeprazole does not bind directly to the AhR but induces CYP1 gene expression *via* modulation of a protein tyrosine kinase-mediated pathway.^47^ GR-*bla* showed no increased CYP activity after incubation with omeprazole and only a minor increase after incubation with B[*a*]P (Figure 2A). CYP activities were slightly increased in AREc32 by both inducers, with the highest CYP activity being 16.59 pmol of resorufin formed per minute per mg_protein_ in the EROD assay after incubation with B[*a*]P. ARE-*bla* showed a stronger induction of the CYP activity by both inducers. The highest CYP activity was measured in the EROD assay after incubation with B[*a*]P with 151.00 pmol of resorufin formed per minute per mg_protein_.

As a comparison with a commonly used metabolization system with increased CYP activity, EROD, EFCOD, and BFCOD assays were also performed with rat liver S9 (Figure 2B). EFCOD activity was lowest (230.56 pmol HFC min^–1^ mg_protein_^–1^) and BFCOD activity was highest (827.29 pmol HFC min^–1^ mg_protein_^–1^) for rat liver S9 (0.1 mg/mL). EROD activity was 695.17 pmol of resorufin min^–1^ mg_protein_^–1^ which is 4.6 times higher than the highest EROD activity of the cell lines. However, it should be noted that S9 from chemically induced rats has a higher metabolic activity than liver fractions from humans and therefore does not simulate the metabolism in humans.^48^

### *In Vitro* Effect Concentrations

All *in vitro* effect concentrations can be found in the Supporting Information (Table S7). Corresponding concentration response curves are shown in Table S8. For five chemicals (ATZ, PCB28, 55DH, IMI, ASP) no cytotoxicity could be determined *via* confluency measurement in all three assays. Nine additional chemicals (CPO, PREG, DEXA, PXS, TAM, HPD, TMX, AZ and 5FU) did not show any cytotoxicity in the AREc32 assay up to the highest tested concentrations, which are also listed in Table S7. No cytotoxicity could be measured *via* confluency for 11 additional chemicals (DMSO, NaAsO_2_, CPO, PREG, DEXA, ETU, CBD, NBNP, TG, TMX, and TETD) in the ARE-*bla* assay and for two additional chemicals (TG and TETD) in the GR-*bla* assay. The chemical with the highest cytotoxicity in all three assays was colchicine (IC_10_ = 3.76 × 10^–9^ M in AREc32, 1.81 × 10^–8^ M in ARE-*bla*, and 2.48 × 10^–9^ M in GR-*bla*). The high toxicity of colchicine can be explained by its antimitotic properties caused by tubulin binding.^49^

For the ARE-*bla* and GR-*bla* assay, cytotoxicity was also measured with the ToxBLAzer reagent (Table S7). Linear fitting of the concentration–response curves was not possible for some chemicals for the ToxBLAzer responses (IZ, 4MIZ, 1MIZ, 1HIZ1P, PPF, 2EIZ, 1E1HIZ, 1VIZ, CARA, HU, and MTX in ARE-*bla* and IZ, 4MIZ, 2MIZ, 1MIZ, 1HIZ1P, 1E1HIZ, 1VIZ, and CARA in GR-*bla*). For these chemicals, cell viability did not decrease with increasing chemical concentration, but appeared to increase. However, the phenomenon only occurred for certain classes of chemicals (*e.g*., imidazoles). A comparison of IC_10,ToxBLAzer_ and IC_10,confluency_ is shown in Figure S8. For some chemicals, cytotoxicity could only be detected with one of the methods (DMSO, NaAsO_2_, CPO, NBNP, TG, 55DH, TETD, and ASP in ARE-*bla* and DEXA, 55DH, HU, and ASP in GR-*bla*). For chemicals for which IC_10_ could be measured with both detection methods, IC_10_ agreed well (Figure S8). IC_10,ToxBLAzer_ values were lower than IC_10,confluency_ values for some chemicals. In the GR-*bla* assay only trichlorfon had a significantly lower IC_10,ToxBLAzer_ (factor of 716). In ARE-*bla* seven chemicals showed differences between both IC_10_ that were higher than a factor of 10. The highest difference was also observed for trichlorfon (factor of 170). Cytotoxicity assays using fluorescent dyes to detect a decrease in metabolic activity are widely used, but have been shown to be prone to artifacts in other studies.^16,50^ However, these methods can be used to detect cell death involving cell swelling (necrosis),^51^ which cannot be detected by confluency-based methods.

### Comparison of Oxidative Stress Response Measured in Two Different Cell Lines

**Figure 3:**
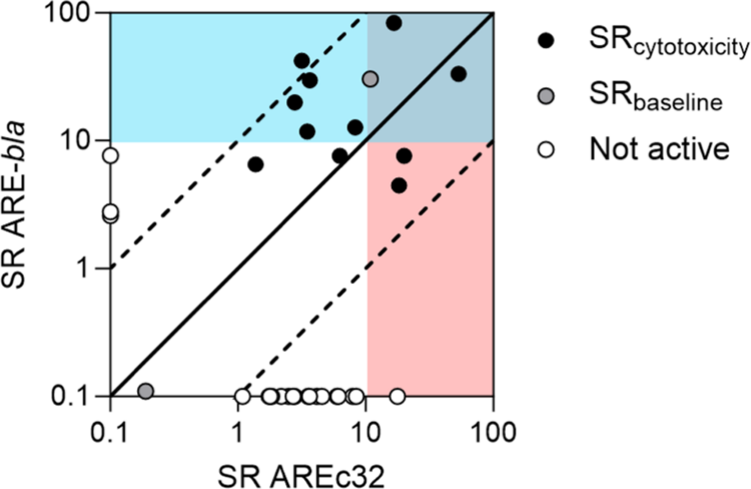
Specificity ratios (SR) of oxidative stress response activation in ARE-*bla* plotted against SR in AREc32. SR_cytotoxicity_ is shown when the cytotoxicity could be determined in both assays (black circles). For chemicals without measured cytotoxicity in at least one assay, SR_baseline_ was used (gray circles). Chemicals which showed oxidative stress response activation only in one assay are indicated with white circles. The solid black line indicates a perfect agreement of SR from both cell lines, and the dashed black lines indicate a deviation by a factor of 10.

AREc32 and ARE-*bla* carry a reporter gene for the oxidative stress response. In AREc32, 31 chemicals activated the oxidative stress response at concentrations below IC_10_. In ARE-*bla*, 17 chemicals activated oxidative stress response. Thirteen chemicals activated oxidative stress response in both assays. Figure 3 shows a comparison of the specificity ratios (SR_cytotoxicity_ or SR_baseline_) of oxidative stress response activation in both assays. The specificity ratio compares the oxidative stress response activation with cytotoxicity or baseline toxicity. SR_baseline_ is shown only if no cytotoxicity could be measured. Five chemicals had specific effects (SR_cytotoxicity_ > 10) in the AREc32 assay (CdCl_2_, 1HIZ1P, GEN, HCY, and oAP) and seven chemicals in the ARE-*bla* assay (NMBAA, NBuAA, NIAA, NDAA, 1HIZ1P, T25D, and oAP). The chemicals with the highest SR_cytotoxicity_ were oAP in AREc32 (SR_cytotoxicity_ = 53.66) and 1HIZ1P in the ARE-*bla* assay (SR_cytotoxicity_ = 83.51) which were also the only chemicals with SR_cytotoxicity_ > 10 in both assays. TMX had SR_baseline_ > 10 in both assays. The inorganic CdCl_2_ activated the reporter gene in AREc32 but was not active at concentrations below cytotoxicity in ARE-*bla*. However, baseline toxicity analysis was not possible for inorganic chemicals, and metabolism also cannot play a role for CdCl_2_.

Seventeen chemicals that showed moderately specific effects (1 < SR < 10) in AREc32 did not activate oxidative stress response below cytotoxicity in ARE-*bla*. The high number of moderately specific chemicals in AREc32 compared with ARE-*bla* is surprising. While 41% of the chemicals that activated oxidative stress response in ARE-*bla* had SR > 10, it was only 16% in AREc32.

The limit of detection (LOD) and limit of quantification (LOQ) were calculated for both assays using eqs 17 and 18 where μ is the average IR and σ is the standard deviation of the unexposed cells.
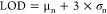
17
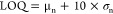
18

The LODs were 0.28 ± 0.08 and 0.24 ± 0.13 for AREc32 and ARE-*bla*, respectively, and LOQs were 0.93 ± 0.28 (AREc32) and 0.79 ± 0.42 (ARE-*bla*), respectively. Even though the LOD and LOQ of the two assays were very similar and the LOQ for ARE-*bla* was slightly lower than that of AREc32, ARE-*bla* showed a higher standard deviation. Figure S9 shows the EC_IR1.5_ values of the reference substance *tert*-butylhydroquinone (tBHQ) for both assays. The average EC_IR1.5_ was 2.33 × 10^–6^ M for AREc32 and a factor of 1.75 higher for ARE-*bla* (4.08 × 10^–6^ M). There was greater variation in the values of the individual plates for ARE-*bla*. It appears that ARE-*bla* has a sensitivity lower than that of AREc32 and therefore cannot detect chemicals that activate the oxidative stress response with only moderate specificity.

GR-*bla* detects glucocorticoid receptor (GR) activation. Only dexamethasone, which is also the reference chemical for the assay, activated GR with an EC_10_ of 3.58 × 10^–10^ M and an SR_cytotoxicity_ of 10.21.

### Comparison of Cytotoxicity Measured in Three Different Cell Lines

If the metabolic activity of the cell lines has an influence on the bioassay results, then differences in cytotoxicity between the cell lines should be apparent. It can be assumed that the highest toxicity is present in GR-*bla*, as there is no metabolic degradation of the test substances and therefore no detoxification. However, for some chemicals, there may also be bioactivation of the test substances, meaning a transformation into reactive metabolites with higher toxicity. As mentioned above, most chemicals without measured IC_10_ were present in ARE-*bla* (16 chemicals) and least chemicals without measured IC_10_ were present in GR-*bla* (7 chemicals) (Table S7).

Dexamethasone showed high cytotoxicity in GR-*bla*, but no cytotoxicity in AREc32 and ARE-*bla*, although the highest tested concentrations were 136,220 (AREc32) or 173,694 (ARE-*bla*) times higher than the IC_10_ values of dexamethasone in GR-*bla*. Dexamethasone is the reference chemical for the GR-*bla* assay and a known GR agonist.^52^ This means that dexamethasone has a specific toxicity mechanism in GR-*bla* that is not present in the other two cell lines. Even if binding to the GR does not lead directly to cell death, a high dose of the receptor agonist restricts the normal function of the reporter gene cell, which ultimately leads to cell death.

Chlorpyrifos-oxon was not cytotoxic in AREc32 and ARE-*bla* and picoxystrobin was not cytotoxic in AREc32. There was a ratio of 140 between AREc32 (highest tested concentration) and GR-*bla* (IC_10_) and a ratio of 279 for ARE-*bla* for chlorpyrifos-oxon. Similarly, the ratio AREc32 (highest tested concentration)/GR-*bla* (IC_10_) was 131 for picoxystrobin. It should be noted that AREc32 and ARE-*bla* both use an assay medium with 10% fetal bovine serum (FBS) and GR-*bla* a medium with only 2% cs-FBS. Particularly in the case of hydrophobic chemicals or protein-reactive chemicals, this can lead to lower bioavailability of the test chemical due to reversible binding to medium proteins or covalent reaction with these proteins.^6,9^ Chlorpyrifos-oxon showed no cytotoxicity in AREc32 and ARE-*bla* although the highest tested concentration was at least 86 times higher than the baseline toxicity. Reactivity toward the proteins of the bioassay medium could explain this observation as protein reactivity of chlorpyrifos-oxon was reported in the literature.^53^

**Figure 4:**
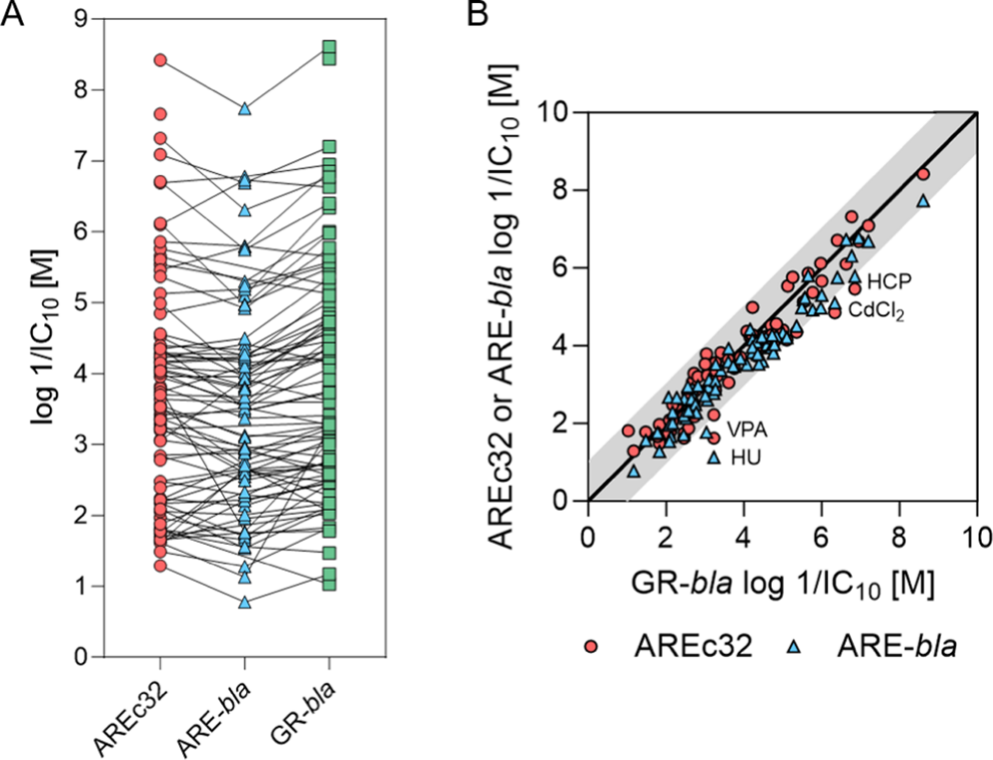
Comparison of cytotoxicity (log 1/IC_10_) of all cell lines. (A) All measured log 1/IC_10_ values from all assays.. (B) Log 1/IC_10_ measured in AREc32 or ARE-*bla* plotted against 1/IC_10_ measured in GR-*bla*. Red circles indicate results for AREc32, blue triangles indicate results for ARE-*bla*, and green squares indicate results for GR-*bla*. The black line in (B) indicates a perfect agreement of the results of the cell lines. The gray area indicates a deviation of a factor of 10. HCP = hexachlorophene, CdCl_2_ = cadmium chloride, VPA = valproic acid, HU = hydroxyurea.

Figure 4A shows that the toxicity of most of the chemicals was slightly lower in ARE-*bla* compared to that of the other two cell lines. An analysis of variance (ANOVA) was performed using Prism 10.1.0. to investigate the significance of the deviation of the log 1/IC_10_ values of the three assays. The log 1/IC_10_ values were normally distributed for all assays as shown in Figure S10. The one-way ANOVA showed no significant difference between the means of the three data sets with a *P*-value of 0.3647.

Figure 4B shows a comparison of the cytotoxicity measured in GR-*bla* that showed no metabolic activity in the EROD, EFCOD and BFCOD assays with the cytotoxicity measured in AREc32 and ARE-*bla*. For the majority of the tested chemicals, IC_10_ from AREc32 and ARE-*bla* agreed within a factor of 10 with the IC_10_ from GR-*bla*. However, the IC_10_ values of hydroxyurea, cadmium chloride and hexachlorophene (AREc32 and ARE-*bla*) and valproic acid (ARE-*bla*) showed more than 10 times deviation from the IC_10_ values of GR-*bla*.

Apparently the higher basal CYP activity of ARE-*bla* compared to GR-*bla* does not influence the cytotoxicity of most chemicals measured in this study, and the chemicals tested do not increase CYP activity. Although the IC_10_ values of most chemicals were higher in ARE-*bla* than in the other two cell lines (Figure 4A), the difference was less than a factor of 10 for most chemicals and larger deviations for few chemicals were not only detected for ARE-*bla* but also for AREc32. AREc32 showed a slight inducibility of metabolic activity, but it was significantly lower than the metabolic activity of ARE-*bla*. Chemicals with largest deviations between GR-*bla* and ARE-*bla* and AREc32 (hydroxyurea, cadmium chloride, and hexachlorophene) are not known to be highly metabolized *in vivo*. None of the chemicals showed a higher toxicity in ARE-*bla* or AREc32 compared to GR-*bla* which speaks against metabolic activation of the chemicals.

### Comparison of Cytotoxicity Measured in Three Different Cell Lines with Baseline Toxicity

Even if there were no clear differences in cytotoxicity between the different cell lines, individual chemicals can be affected by cellular metabolism. To identify these chemicals, the measured cytotoxicity can be compared with the predicted minimum toxicity (baseline toxicity). If the measured toxicity is lower than the baseline toxicity (TR < 0.1), this may indicate a loss of the chemical. However, this approach only identifies loss of baseline toxicants. Experimental artifacts or loss of specifically acting chemicals with TR > 10 cannot be identified with this approach, as they might just have a reduced TR or be wrongly classified as baseline toxicants.

**Figure 5:**
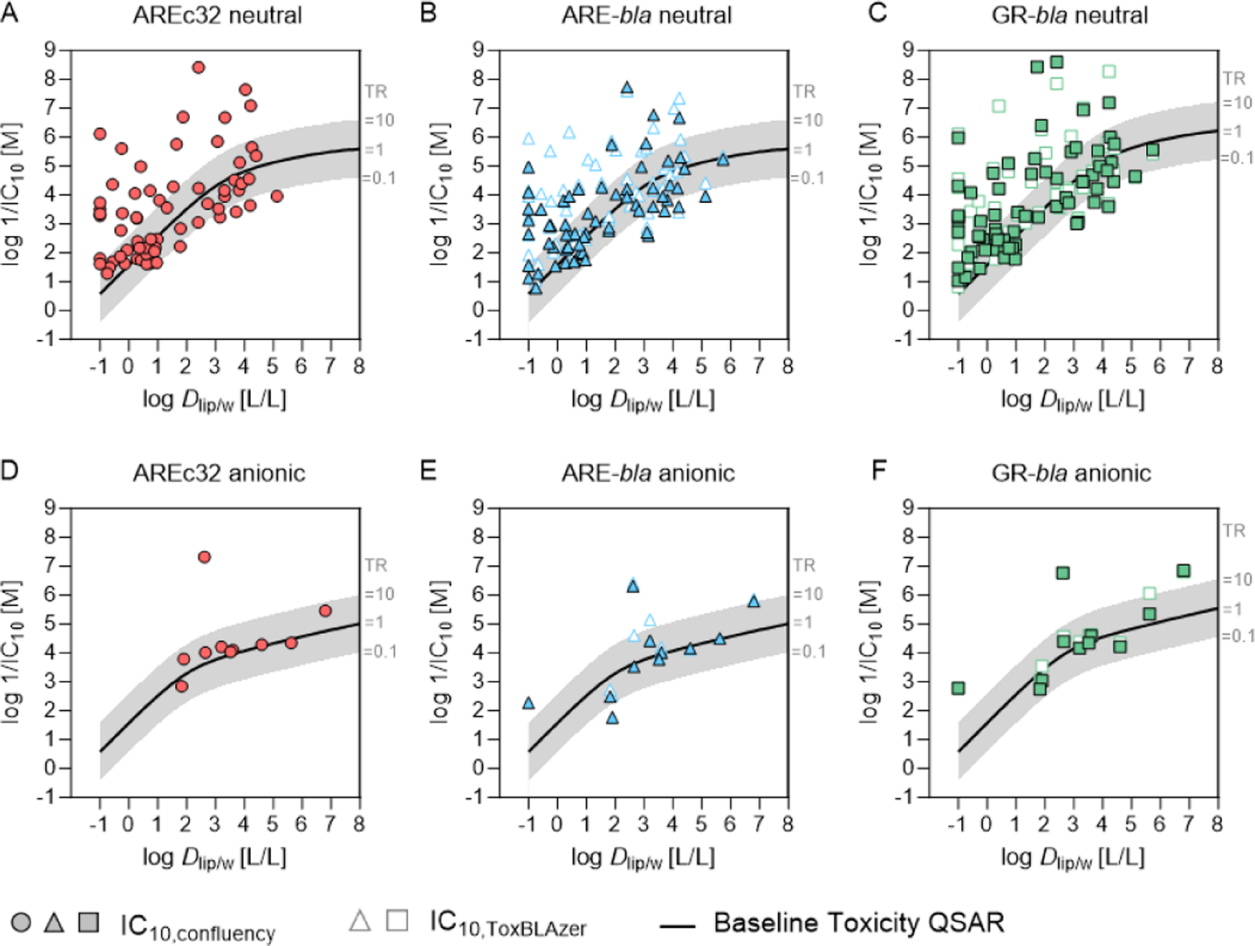
Cytotoxicity of test chemicals compared to baseline toxicity. Logarithmic reciprocal IC_10,confluency_ and logarithmic reciprocal IC_10,ToxBLAzer_ were plotted against the logarithmic liposome–water distribution ratios (log  *D*_lip/w_) of the test chemicals. The black line indicates IC_10,baseline_, and the gray area indicates a toxic ratio (TR) between 0.1 and 10. (A, D) Data from AREc32 assay for neutral (A) and anionic chemicals (D). (B, E) Data from ARE-*bla* assay for neutral (B) and anionic chemicals (E). (C, F) Data from GR-*bla* assay for neutral (C) and anionic chemicals (F).

The measured cytotoxicity of the chemicals was compared with baseline toxicity using the newly defined baseline IC_10_ based on a critical membrane concentration of 26 mmol/L_lip_ for neutral and anionic chemicals and for the media with either 10% FBS or 2% cs-FBS. Figure 5 shows the logarithmic reciprocal IC_10_ values of all chemicals plotted against log *D*_lip/w_. Four chemicals (CdCl_2_, As_2_O_3_, NaAsO_2_, and TEtT) were metals or inorganic chemical without measured *D*_lip/w_, so no IC_10,baseline_ could be predicted.

From the remaining 90 chemicals, 27 showed specific toxicity in AREc32 (26 neutral and one anionic chemical, Figure 5A,5D), and 22 showed specific toxicity in ARE-*bla* (19 neutral and three anionic chemicals, Figure 5B,5E) when using IC_10,confluency_ and 32 chemicals (29 neutral and three anionic chemicals, Figure 5B,5E) when using IC_10,ToxBLAzer_. In GR-*bla*, 28 chemicals showed specific toxicity (25 neutral and three anionic chemicals, Figure 5C,5F) when using IC_10,confluency_ and 27 chemicals (23 neutral and four anionic chemicals, Figure 5C,F) when using IC_10,ToxBLAzer_. The chemical with the highest TR in all assays was azacytidine, a cytostatic drug used in cancer therapy. Azacytidine exerts toxic effects by intercalating into DNA and inhibition of DNA methyltransferases which ultimately leads to chromosomal instability and cell death.^54^ The majority of the chemicals were baseline toxicants, and the new baseline toxicity QSAR could predict baseline toxicity for both neutral chemicals and anionic chemicals.

For five chemicals (AREc32 and GR-*bla*) or six chemicals (ARE-*bla*), calculated TR values were below 0.1. Valproic acid (VPA) was the only anionic chemical with a TR < 0.1, but only in ARE-*bla*. VPA is an anticonvulsant drug which is rapidly metabolized in the liver,^55^ so possibly metabolic degradation could be responsible for the low TR in ARE-*bla* which is also confirmed by the comparison with GR-*bla* (Figure 4) which showed a significantly lower IC_10_ for VPA.

For other chemicals with TR < 0.1, low TRs were observed in all assays suggesting that cellular metabolism was not involved. It is more likely to be an artifact as baseline toxicity is the minimal toxicity a chemical can have. TR below 0.1 can be caused by experimental errors, *e.g*., precipitation of the chemical or by wrong physicochemical properties as input parameters for the baseline toxicity QSAR. *D*_lip/w_ of all chemicals with TR < 0.1 were predicted with LSER or QSAR models, so inaccurate *D*_lip/w_ could be responsible for this artifact. Also, chemical loss processes like volatilization or degradation can lead to TR < 0.1. Chemicals with TR < 0.1 in at least one assay were EPP, NMA, TDM, CP, DPA, VPA, and CBZ.

There are no indications of possible abiotic instability in the literature for most of the chemicals. Only ethoprophos (EPP) and chlorpyrifos (CP) could possibly be hydrolyzed in the course of bioassay like it has been observed for other organophosphates.^9,56^*N*-Methylaniline (NMA) and CP have predicted water–air partitioning constants of 3.62 × 10^–4^ and 1.19 × 10^–4^, so volatilization could be responsible for the low TR of these two chemicals, especially in bioassay medium with a low FBS content. In order to clarify the cause of the low TR for these chemicals, the concentration in the bioassay should be determined experimentally, and possible metabolites should be identified.

## Conclusions

The revised baseline toxicity QSAR is a clear improvement over previous models as it extends the QSAR to more hydrophilic chemicals than previously published studies,^8,17^ down to a log *D*_lip/w_ of −1, and includes specifically anionic chemicals. In protein-rich medium, two different QSAR lines apply for neutral and anionic chemicals due to the stronger binding of anions to proteins. The model for neutral chemicals (eqs 13 and 15) is also applicable to cations and zwitterions, provided the log *D*_lip/w_ accounts for speciation.^17^ The revised baseline toxicity QSAR is shifted 0.42 log units higher (lower IC_10,baseline_) than the previously used QSAR (Figure S11), which means that some chemicals that were previously classified as moderately specific will now still be classified as baseline toxicants.

The improved baseline toxicity QSAR can be used to distinguish between baseline toxicants and specifically acting chemicals. In the present study, the chemicals with the most specific cytotoxicity (highest TR) were azacytidine and colchicine in all assays. Although there were seven chemicals with TR < 0.1, they amounted to less than 10% of the tested chemicals. The reason for this artifact could not fully be explained, but it is likely that experimental artifacts or incorrectly predicted physicochemical properties are a major cause of the inaccurate prediction of baseline toxicity. TR < 0.1 can also be an indication of chemical loss processes, but only for baseline toxicants and cannot differentiate between loss of specifically acting chemicals and reduction of their effect. For this reason, experimental measurement of the freely dissolved or total concentration in the bioassay should be performed to ensure stable chemical exposure during the bioassay.

**Figure 6:**
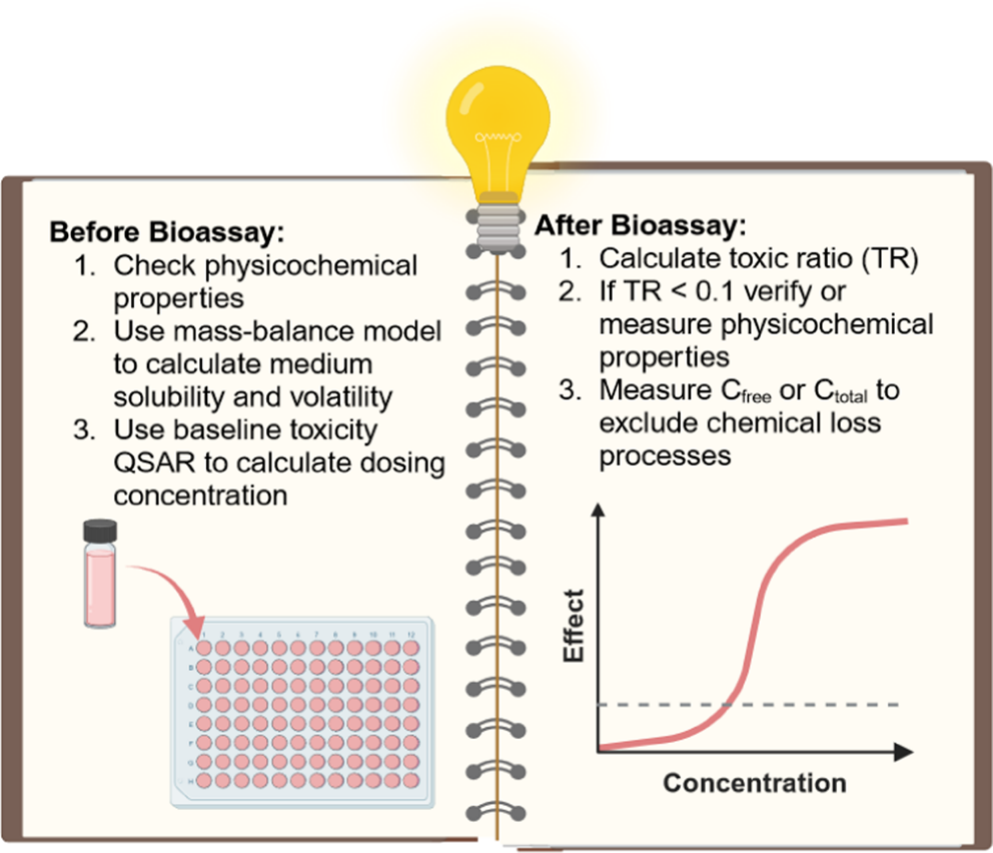
Testing strategies for single chemical screening in high-throughput *in vitro* bioassays.

This study has shown that a number of points (Figure 6) need to be considered before and after the high-throughput screening of single chemicals in order to obtain reliable results. A careful consideration of the physicochemical properties of the chemicals can exclude chemical precipitation or volatilization during the assay. Mass-balance models can be used to calculate solubility in bioassay medium and medium-air partitioning constants to identify possibly volatile chemicals.^8,57^ Experimental *D*_lip/w_ of the chemical will give a better prediction of baseline toxicity, because predicted values are often subject to uncertainty, especially if they are based on predicted *K*_ow_ or if speciation is incorrectly measured or predicted. A dosing concentration of 3 times the calculated baseline toxicity is recommended if this is below medium solubility. After the experiment, TR can be calculated to identify chemicals with specific modes of action, but also to identify chemicals with experimental issues such as precipitation, volatilization, or degradation. Effect concentrations of problematic chemicals should be reevaluated, and loss processes should be experimentally excluded if these data are to be used for chemical risk assessment.

Another aim of this study was to evaluate the impact of the metabolic activity of reporter gene cell lines on *in vitro* results. The metabolic activity of the three reporter gene cell lines of this study was found to be low, as already described in the literature.^23^ The highest activity was observed for the ARE-*bla* cell line, which is explained by its liver origin. For the 94 chemicals tested in this study, no clear difference in cytotoxicity in the three different cell lines could be identified, even though a higher metabolic activity of the HepG2-based ARE-*bla* cell line was measured. Although ARE-*bla* showed a slightly lower chemical susceptibility with slightly higher IC_10_ values for the majority of chemicals compared to the other two cell lines, this difference was found to be not significant. Nevertheless, ARE-*bla* showed a lower sensitivity for the activation of the oxidative stress response, as a much smaller number of chemicals showed activity in ARE-*bla* than in AREc32. The more sensitive AREc32 assay should preferably be used for a conservative risk assessment as chemicals with less specific effects can also be identified. Such chemicals may not pose a high risk as individual substances, but in mixtures with other chemicals with the same mode of action, they can contribute to mixture effects.^58^

Although strong CYP inducers, such as benzo[*a*]pyrene, were able to induce metabolic enzymes in ARE-*bla* and AREc32, metabolism rates must be high enough to significantly reduce the internal chemical concentration and, thus, alter the measured toxicity. Since the cell lines showed a low basal CYP activity and even after chemical induction it was almost 5 times lower than the CYP activity of rat liver S9, metabolic activity of the three cell lines used is apparently not sufficient to influence the cytotoxicity of the chemicals of the present study or metabolites and parent chemicals have similar cytotoxicity. It has been shown that despite the different metabolic activity of the three cell lines, there is no systematic change in the measured *in vitro* effects. This result is positive, as it indicates a good comparability of the *in vitro* data from different cell lines. However, if metabolism in the *in vitro* system is desired to study the toxic effects of metabolites that might be formed *in vivo*, a separate metabolism system such as liver microsomes, S9 fractions or purified enzymes must be added to the chemicals either before or during the *in vitro* bioassay.^20^

## Supporting Information Available

Chemicals for baseline toxicity QSAR development with effect concentrations and concentration response curves; details on CYP inducers and metabolic characterization; comparison of confluency measurement and ToxBLAzer measurement for cytotoxicity determination; oxidative stress response activation of reference compound tBHQ, normal distribution of cytotoxicity; and comparison of newly defined baseline toxicity QSAR with QSAR from the literature (PDF)
Test chemicals and effect concentrations and concentration response curves of all chemicals and assays (XLSX)

## References

[ref1] Van Norman G. A. Limitations of Animal Studies for Predicting Toxicity in Clinical Trials: Is it Time to Rethink Our Current Approach?. JACC Basic Transl. Sci. 2019, 4 (7), 845–854. 10.1016/j.jacbts.2019.10.008. 31998852 PMC6978558

[ref2] Isaacs K. K.; Egeghy P.; Dionisio K. L.; Phillips K. A.; Zidek A.; Ring C.; Sobus J. R.; Ulrich E. M.; Wetmore B. A.; Williams A. J.; Wambaugh J. F. The chemical landscape of high-throughput new approach methodologies for exposure. J. Exposure Sci. Environ. Epidemiol. 2022, 32 (6), 820–832. 10.1038/s41370-022-00496-9. PMC9882966 36435938

[ref3] Schmeisser S.; Miccoli A.; von Bergen M.; Berggren E.; Braeuning A.; Busch W.; Desaintes C.; Gourmelon A.; Grafström R.; Harrill J.; Hartung T.; Herzler M.; Kass G. E. N.; Kleinstreuer N.; Leist M.; Luijten M.; Marx-Stoelting P.; Poetz O.; van Ravenzwaay B.; Roggeband R.; Rogiers V.; Roth A.; Sanders P.; Thomas R. S.; Marie Vinggaard A.; Vinken M.; van de Water B.; Luch A.; Tralau T. New approach methodologies in human regulatory toxicology - Not if, but how and when!. Environ. Int. 2023, 178, 108082 10.1016/j.envint.2023.108082. 37422975 PMC10858683

[ref4] van der Zalm A. J.; Barroso J.; Browne P.; Casey W.; Gordon J.; Henry T. R.; Kleinstreuer N. C.; Lowit A. B.; Perron M.; Clippinger A. J. A framework for establishing scientific confidence in new approach methodologies. Arch. Toxicol. 2022, 96 (11), 2865–2879. 10.1007/s00204-022-03365-4. 35987941 PMC9525335

[ref5] Freedman J.; The Precision Toxicology initiative. Toxicol. Lett. 2023, 383, 33–42. 10.1016/j.toxlet.2023.05.004. 37211341

[ref6] Henneberger L.; Mühlenbrink M.; Fischer F. C.; Escher B. I. C18-Coated Solid-Phase Microextraction Fibers for the Quantification of Partitioning of Organic Acids to Proteins, Lipids, and Cells. Chem. Res. Toxicol. 2019, 32 (1), 168–178. 10.1021/acs.chemrestox.8b00249. 30585484

[ref7] Fischer F. C.; Cirpka O. A.; Goss K. U.; Henneberger L.; Escher B. I. Application of Experimental Polystyrene Partition Constants and Diffusion Coefficients to Predict the Sorption of Neutral Organic Chemicals to Multiwell Plates in in Vivo and in Vitro Bioassays. Environ. Sci. Technol. 2018, 52 (22), 13511–13522. 10.1021/acs.est.8b04246. 30298728

[ref8] Escher B. I.; Glauch L.; König M.; Mayer P.; Schlichting R. Baseline Toxicity and Volatility Cutoff in Reporter Gene Assays Used for High-Throughput Screening. Chem. Res. Toxicol. 2019, 32 (8), 1646–1655. 10.1021/acs.chemrestox.9b00182. 31313575

[ref9] Huchthausen J.; Henneberger L.; Mälzer S.; Nicol B.; Sparham C.; Escher B. I. High-Throughput Assessment of the Abiotic Stability of Test Chemicals in In Vitro Bioassays. Chem. Res. Toxicol. 2022, 35 (5), 867–879. 10.1021/acs.chemrestox.2c00030. 35394761

[ref10] Fischer F. C.; Abele C.; Henneberger L.; Klüver N.; König M.; Mühlenbrink M.; Schlichting R.; Escher B. I. Cellular Metabolism in High-Throughput In Vitro Reporter Gene Assays and Implications for the Quantitative In Vitro-In Vivo Extrapolation. Chem. Res. Toxicol. 2020, 33 (7), 1770–1779. 10.1021/acs.chemrestox.0c00037. 32227843

[ref11] Henneberger L.; Mühlenbrink M.; König M.; Schlichting R.; Fischer F. C.; Escher B. I. Quantification of freely dissolved effect concentrations in in vitro cell-based bioassays. Arch. Toxicol. 2019, 93 (8), 2295–2305. 10.1007/s00204-019-02498-3. 31230094

[ref12] Huchthausen J.; König M.; Escher B. I.; Henneberger L. Experimental exposure assessment for in vitro cell-based bioassays in 96- and 384-well plates. Front. Toxicol. 2023, 5, 1221625 10.3389/ftox.2023.1221625. 37564394 PMC10411540

[ref13] Fischer F. C.; Henneberger L.; König M.; Bittermann K.; Linden L.; Goss K. U.; Escher B. I. Modeling Exposure in the Tox21 in Vitro Bioassays. Chem. Res. Toxicol. 2017, 30 (5), 1197–1208. 10.1021/acs.chemrestox.7b00023. 28316234

[ref14] Armitage J. M.; Sangion A.; Parmar R.; Looky A. B.; Arnot J. A. Update and Evaluation of a High-Throughput In Vitro Mass Balance Distribution Model: IV-MBM EQP v2.0. Toxics 2021, 9 (11), 31 10.3390/toxics9110315. 34822706 PMC8625852

[ref15] Verhaar H. J. M.; Ramos E. U.; Hermens J. L. M. Classifying environmental pollutants. 2. Separation of class 1 (baseline toxicity) and class 2 (’polar narcosis’) type compounds based on chemical descriptors. J. Chem. 1996, 10 (2), 149–162. 10.1002/(sici)1099-128x(199603)10:23.0.co;2-f.

[ref16] Escher B. I.; Henneberger L.; König M.; Schlichting R.; Fischer F. C. Cytotoxicity Burst? Differentiating Specific from Nonspecific Effects in Tox21 in Vitro Reporter Gene Assays. Environ. Health Perspect. 2020, 128 (7), 77007 10.1289/EHP6664. 32700975 PMC7377237

[ref17] Lee J.; Braun G.; Henneberger L.; König M.; Schlichting R.; Scholz S.; Escher B. I. Critical Membrane Concentration and Mass-Balance Model to Identify Baseline Cytotoxicity of Hydrophobic and Ionizable Organic Chemicals in Mammalian Cell Lines. Chem. Res. Toxicol. 2021, 34 (9), 2100–2109. 10.1021/acs.chemrestox.1c00182. 34357765

[ref18] Maeder V.; Escher B. I.; Scheringer M.; Hungerbühler K. Toxic ratio as an indicator of the intrinsic toxicity in the assessment of persistent, bioaccumulative, and toxic chemicals. Environ. Sci. Technol. 2004, 38 (13), 3659–3666. 10.1021/es0351591. 15296318

[ref19] Qin W.; Henneberger L.; Glüge J.; König M.; Escher B. I. Baseline toxicity model to identify the specific and non-specific effects of per- and polyfluoroalkyl substances in cell-based bioassays. Environ. Sci. Technol. 2024, 58 (13), 5727–5738. 10.1021/acs.est.3c09950. 38394616 PMC10993398

[ref20] Coecke S.; Ahr H.; Blaauboer B. J.; Bremer S.; Casati S.; Castell J.; Combes R.; Corvi R.; Crespi C. L.; Cunningham M. L.; Elaut G.; Eletti B.; Freidig A.; Gennari A.; Ghersi-Egea J. F.; Guillouzo A.; Hartung T.; Hoet P.; Ingelman-Sundberg M.; Munn S.; Janssens W.; Ladstetter B.; Leahy D.; Long A.; Meneguz A.; Monshouwer M.; Morath S.; Nagelkerke F.; Pelkonen O.; Ponti J.; Prieto P.; Richert L.; Sabbioni E.; Schaack B.; Steiling W.; Testai E.; Vericat J. A.; Worth A. Metabolism: a bottleneck in in vitro toxicological test development. The report and recommendations of ECVAM workshop 54. Altern. Lab. Anim. 2006, 34 (1), 49–84. 10.1177/026119290603400113. 16522150

[ref21] Ooka M.; Lynch C.; Xia M. Application of In Vitro Metabolism Activation in High-Throughput Screening. Int. J. Mol. Sci. 2020, 21 (21), 8182 10.3390/ijms21218182. 33142951 PMC7663506

[ref22] Hopperstad K.; Deisenroth C. Development of a bioprinter-based method for incorporating metabolic competence into high-throughput in vitro assays. Front. Toxicol. 2023, 5, 1196245 10.3389/ftox.2023.1196245. 37215384 PMC10192685

[ref23] Qu W.; Crizer D. M.; DeVito M. J.; Waidyanatha S.; Xia M.; Houck K.; Ferguson S. S. Exploration of xenobiotic metabolism within cell lines used for Tox21 chemical screening. Toxicol. In Vitro. 2021, 73, 105109 10.1016/j.tiv.2021.105109. 33609632 PMC10838150

[ref24] Choi J. M.; Oh S. J.; Lee S. Y.; Im J. H.; Oh J. M.; Ryu C. S.; Kwak H. C.; Lee J. Y.; Kang K. W.; Kim S. K. HepG2 cells as an in vitro model for evaluation of cytochrome P450 induction by xenobiotics. Arch. Pharm. Res. 2015, 38 (5), 691–704. 10.1007/s12272-014-0502-6. 25336106

[ref25] Iwanari M.; Nakajima M.; Kizu R.; Hayakawa K.; Yokoi T. Induction of CYP1A1, CYP1A2, and CYP1B1 mRNAs by nitropolycyclic aromatic hydrocarbons in various human tissue-derived cells: chemical-, cytochrome P450 isoform-, and cell-specific differences. Arch. Toxicol. 2002, 76 (5–6), 287–298. 10.1007/s00204-002-0340-z. 12107646

[ref26] Fischer F. C.; Abele C.; Droge S. T. J.; Henneberger L.; König M.; Schlichting R.; Scholz S.; Escher B. I. Cellular Uptake Kinetics of Neutral and Charged Chemicals in in Vitro Assays Measured by Fluorescence Microscopy. Chem. Res. Toxicol. 2018, 31 (8), 646–657. 10.1021/acs.chemrestox.8b00019. 29939727

[ref27] Attene-Ramos M. S.; Miller N.; Huang R.; Michael S.; Itkin M.; Kavlock R. J.; Austin C. P.; Shinn P.; Simeonov A.; Tice R. R.; Xia M. The Tox21 robotic platform for the assessment of environmental chemicals-from vision to reality. Drug Discovery Today 2013, 18 (15–16), 716–723. 10.1016/j.drudis.2013.05.015. 23732176 PMC3771082

[ref28] Wang X. J.; Hayes J. D.; Wolf C. R. Generation of a stable antioxidant response element-driven reporter gene cell line and its use to show redox-dependent activation of nrf2 by cancer chemotherapeutic agents. Cancer Res. 2006, 66 (22), 10983–10994. 10.1158/0008-5472.CAN-06-2298. 17108137

[ref29] Shukla S. J.; Huang R.; Simmons S. O.; Tice R. R.; Witt K. L.; Vanleer D.; Ramabhadran R.; Austin C. P.; Xia M. Profiling environmental chemicals for activity in the antioxidant response element signaling pathway using a high throughput screening approach. Environ. Health Perspect. 2012, 120 (8), 1150–1156. 10.1289/ehp.1104709. 22551509 PMC3440086

[ref30] Wilkinson J. M.; Hayes S.; Thompson D.; Whitney P.; Bi K. Compound profiling using a panel of steroid hormone receptor cell-based assays. J. Biomol. Screening 2008, 13 (8), 755–765. 10.1177/1087057108322155. 18753690

[ref31] Kennedy S. W.; Lorenzen A.; James C. A.; Collins B. T. Ethoxyresorufin-O-deethylase and porphyrin analysis in chicken embryo hepatocyte cultures with a fluorescence multiwell plate reader. Anal. Biochem. 1993, 211 (1), 102–112. 10.1006/abio.1993.1239. 8323021

[ref32] DeLuca J. G.; Dysart G. R.; Rasnick D.; Bradley M. O. A direct, highly sensitive assay for cytochrome P-450 catalyzed O-deethylation using a novel coumarin analog. Biochem. Pharmacol. 1988, 37 (9), 1731–1739. 10.1016/0006-2952(88)90436-4. 3259881

[ref33] Renwick A. B.; Surry D.; Price R. J.; Lake B. G.; Evans D. C. Metabolism of 7-benzyloxy-4-trifluoromethyl-coumarin by human hepatic cytochrome P450 isoforms. Xenobiotica 2000, 30 (10), 955–969. 10.1080/00498250050200113. 11315104

[ref34] Niu L.; Henneberger L.; Huchthausen J.; Krauss M.; Ogefere A.; Escher B. I. pH-Dependent Partitioning of Ionizable Organic Chemicals between the Silicone Polymer Polydimethylsiloxane (PDMS) and Water. ACS Environ. Au 2022, 2 (3), 253–262. 10.1021/acsenvironau.1c00056. 37102138 PMC10114720

[ref35] König M.; Escher B. I.; Neale P. A.; Krauss M.; Hilscherova K.; Novak J.; Teodorovic I.; Schulze T.; Seidensticker S.; Hashmi M. A. K.; Ahlheim J.; Brack W. Impact of untreated wastewater on a major European river evaluated with a combination of in vitro bioassays and chemical analysis. Environ. Pollut. 2017, 220 (Pt B), 1220–1230. 10.1016/j.envpol.2016.11.011. 27884472

[ref36] Neale P. A.; Altenburger R.; Ait-Aissa S.; Brion F.; Busch W.; de Aragao Umbuzeiro G.; Denison M. S.; Du Pasquier D.; Hilscherova K.; Hollert H.; Morales D. A.; Novak J.; Schlichting R.; Seiler T. B.; Serra H.; Shao Y.; Tindall A. J.; Tollefsen K. E.; Williams T. D.; Escher B. I. Development of a bioanalytical test battery for water quality monitoring: Fingerprinting identified micropollutants and their contribution to effects in surface water. Water Res. 2017, 123, 734–750. 10.1016/j.watres.2017.07.016. 28728110

[ref37] Escher B. I.; Dutt M.; Maylin E.; Tang J. Y.; Toze S.; Wolf C. R.; Lang M. Water quality assessment using the AREc32 reporter gene assay indicative of the oxidative stress response pathway. J. Environ. Monit. 2012, 14 (11), 2877–2885. 10.1039/c2em30506b. 23032559

[ref38] Wezel A. P. v.; Opperhuizen A. Narcosis due to environmental pollutants in aquatic organisms: residue-based toxicity, mechanisms, and membrane burdens. Crit. Rev. Toxicol. 1995, 25 (3), 255–279. 10.3109/10408449509089890. 7576154

[ref39] Huchthausen J.; Mühlenbrink M.; König M.; Escher B. I.; Henneberger L. Experimental Exposure Assessment of Ionizable Organic Chemicals in In Vitro Cell-Based Bioassays. Chem. Res. Toxicol. 2020, 33 (7), 1845–1854. 10.1021/acs.chemrestox.0c00067. 32368900

[ref40] Ulrich N.; Endo S.; Brown T. N.; Watanabe N.; Bronner G.; Abraham M. H.; Goss K.-U. UFZ-LSER database v 3.2.1 [Internet], 2017. http://www.ufz.de/lserd.

[ref41] Endo S.; Escher B. I.; Goss K.-U. Capacities of Membrane Lipids to Accumulate Neutral Organic Chemicals. Environ. Sci. Technol. 2011, 45 (14), 5912–5921. 10.1021/es200855w. 21671592

[ref42] Endo S.; Goss K. U. Serum albumin binding of structurally diverse neutral organic compounds: data and models. Chem. Res. Toxicol. 2011, 24 (12), 2293–2301. 10.1021/tx200431b. 22070391

[ref43] Escher B. I.; Neale P. A.; Villeneuve D. L. The advantages of linear concentration-response curves for in vitro bioassays with environmental samples. Environ. Toxicol. Chem. 2018, 37 (9), 2273–2280. 10.1002/etc.4178. 29846006 PMC6150494

[ref44] Escher B. I.; Schwarzenbach R. P. Mechanistic studies on baseline toxicity and uncoupling of organic compounds as a basis for modeling effective membrane concentrations in aquatic organisms. Aquat. Sci. 2002, 64 (1), 20–35. 10.1007/s00027-002-8052-2.

[ref45] Escher B. I.; Baumer A.; Bittermann K.; Henneberger L.; König M.; Kühnert C.; Klüver N. General baseline toxicity QSAR for nonpolar, polar and ionisable chemicals and their mixtures in the bioluminescence inhibition assay with Aliivibrio fischeri. Environ. Sci.: Processes Impacts 2017, 19 (3), 414–428. 10.1039/C6EM00692B. 28197603

[ref46] Shah U. K.; Seager A. L.; Fowler P.; Doak S. H.; Johnson G. E.; Scott S. J.; Scott A. D.; Jenkins G. J. A comparison of the genotoxicity of benzo[a]pyrene in four cell lines with differing metabolic capacity. Mutat. Res., Genet. Toxicol. Environ. Mutagen. 2016, 808, 8–19. 10.1016/j.mrgentox.2016.06.009. 27637481

[ref47] Kikuchi H.; Hossain A. Signal transduction-mediated CYP1A1 induction by omeprazole in human HepG2 cells. Exp. Toxicol. Pathol. 1999, 51 (4), 342–346. 10.1016/S0940-2993(99)80018-9. 10445394

[ref48] Cox J. A.; Fellows M. D.; Hashizume T.; White P. A. The utility of metabolic activation mixtures containing human hepatic post-mitochondrial supernatant (S9) for in vitro genetic toxicity assessment. Mutagenesis 2016, 31 (2), 117–130. 10.1093/mutage/gev082. 26712374

[ref49] Sackett D. L.; Varma J. K. Molecular mechanism of colchicine action: induced local unfolding of beta-tubulin. Biochemistry 1993, 32 (49), 13560–13565. 10.1021/bi00212a023. 8257691

[ref50] Hsieh J. H.; Huang R.; Lin J. A.; Sedykh A.; Zhao J.; Tice R. R.; Paules R. S.; Xia M.; Auerbach S. S. Real-time cell toxicity profiling of Tox21 10K compounds reveals cytotoxicity dependent toxicity pathway linkage. PLoS One 2017, 12 (5), e0177902 10.1371/journal.pone.0177902. 28531190 PMC5439695

[ref51] Martin S. J.; Henry C. M. Distinguishing between apoptosis, necrosis, necroptosis and other cell death modalities. Methods 2013, 61 (2), 87–89. 10.1016/j.ymeth.2013.06.001. 23768793

[ref52] Li H.; Qian W.; Weng X.; Wu Z.; Li H.; Zhuang Q.; Feng B.; Bian Y. Glucocorticoid receptor and sequential P53 activation by dexamethasone mediates apoptosis and cell cycle arrest of osteoblastic MC3T3-E1 cells. PLoS One 2012, 7 (6), e37030 10.1371/journal.pone.0037030. 22719835 PMC3375272

[ref53] Schopfer L. M.; Lockridge O. Mass Spectrometry Identifies Isopeptide Cross-Links Promoted by Diethylphosphorylated Lysine in Proteins Treated with Chlorpyrifos Oxon. Chem. Res. Toxicol. 2019, 32 (4), 762–772. 10.1021/acs.chemrestox.9b00001. 30844252

[ref54] Raj K.; Mufti G. J. Azacytidine (Vidaza(R)) in the treatment of myelodysplastic syndromes. Ther. Clin. Risk Manage. 2006, 2 (4), 377–388. 10.2147/tcrm.2006.2.4.377. PMC1936359 18360650

[ref55] Ghodke-Puranik Y.; Thorn C. F.; Lamba J. K.; Leeder J. S.; Song W.; Birnbaum A. K.; Altman R. B.; Klein T. E. Valproic acid pathway: pharmacokinetics and pharmacodynamics. Pharmacogenet. Genomics 2013, 23 (4), 236–241. 10.1097/FPC.0b013e32835ea0b2. 23407051 PMC3696515

[ref56] Silva V. B.; Orth E. S. Structure-Reactivity Insights on the Alkaline Hydrolysis of Organophosphates: Non-Leaving and Leaving Group Effects in a Bilinear Brønsted-Like Relationship. ChemPhysChem 2023, 24 (6), e202200612 10.1002/cphc.202200612. 36326485

[ref57] Fischer F. C.; Henneberger L.; Schlichting R.; Escher B. I. How To Improve the Dosing of Chemicals in High-Throughput in Vitro Mammalian Cell Assays. Chem. Res. Toxicol. 2019, 32 (8), 1462–1468. 10.1021/acs.chemrestox.9b00167. 31328914

[ref58] Escher B.; Braun G.; Zarfl C. Exploring the Concepts of Concentration Addition and Independent Action Using a Linear Low-Effect Mixture Model. Environ. Toxicol. Chem. 2020, 39 (12), 2552–2559. 10.1002/etc.4868. 32897547

