# Supplementary material for: Effects of Chemicals in Reporter Gene Bioassays with Different Metabolic Activities Compared to Baseline Toxicity
Source: Chem Res Toxicol. 2024 Apr 23;37(5):744–56. doi: 10.1021/acs.chemrestox.4c00017 (PMC11110108; doi:10.1021/acs.chemrestox.4c00017)
Supplement: Supplementary file 1 — tx4c00017_si_001.pdf [file tx4c00017_si_001.pdf]

Supporting information for
Effects of chemicals in reporter-gene bioassays with different metabolic activity compared to baseline toxicity

Julia Huchthausen[†], Jenny Braasch[†], Beate I. Escher[†,§], Maria König[†], Luise Henneberger[†,*]

[†] Helmholtz Centre for Environmental Research – UFZ, Department of Cell Toxicology, Permoserstr. 15, 04318 Leipzig, Germany

[§] Eberhard Karls University Tübingen, Environmental Toxicology, Department of Geosciences, 72076 Tübingen, Germany

* Corresponding author
luise.henneberger@ufz.de

Table of content

Excel Table S1: Chemical information and physicochemical properties of the test chemicals.................... 3

Text S1: Measurement of logarithmic octanol-water partition ratios (log K_{ow}) ratios of the test chemicals. 3

Table S2: Hydrophilic chemicals for baseline QSAR.. 4

Table S3: Protein and lipid contents of the bioassay media used for the three assays taken from Qin et al. (2024). 4

Text S2: Optimization of EROD, EFCOD and BFCOD assay. 4

Figure S1: Concentration-response curves (CRC) of the CYP inducers in the AREc32 assay. 5

Figure S2: Concentration-response curves (CRC) of the CYP inducers in the ARE-*bla* assay. 6

Figure S3: Concentration-response curves (CRC) of the CYP inducers in the GR-*bla* assay...................... 6

Figure S4: Optimization of 7-ethoxyresorufin (ETX), 7-ethoxy-4-trifluoromethylcoumarin (EFC) and 7-benzyloxy-4-trifluoromethylcoumarin (BFC) concentrations. 7

Table S4: Effect concentrations of CYP inducers... 7

Figure S5: Concentration response curves (CRC) for hydrophilic chemicals in the AREc32 assay............ 8

Figure S6: Concentration response curves (CRC) for hydrophilic chemicals in the ARE-*bla* assay. 9

Figure S7: Concentration response curves (CRC) for hydrophilic chemicals in the GR-*bla* assay. 10

Table S5: Concentration causing a reduction of cell viability of 10 % (IC_{10}) for hydrophilic chemicals. .. 11

Table S6: Freely dissolved concentration causing a reduction of cell viability of 10 % ($IC_{10,free}$) and logarithmic liposome-water distribution ratios for AREc32 assay for 14 ionizable organic chemicals and caffeine from Huchthausen et al. (2020). 11

Excel Table S7: *In vitro* effect concentrations of all chemicals in AREc32, ARE-*bla* and GR-*bla* assay.. 12

Excel Table S8: Concentration-response curves (CRC) of all chemicals in all assays for cytotoxicity, ToxBLAzer cytotoxicity, oxidative stress response activation and glucocorticoid receptor (GR) activation. 12

Figure S8: Comparison of cytotoxicity determined with confluency measurement (Confluency log $1/IC_{10}$) or with the ToxBLAzer™ reagent (ToxBLAzer log $1/IC_{10}$)... 12

Figure S9: Oxidative stress response activation ($EC_{IR1.5}$) of reference compound *tert*-butylhydroquinone (tBHQ) for AREc32 and ARE-*bla* assay. 13

Figure S10: Normal distribution of bioassay cytotoxicity data (log $1/IC_{10}$). 13

Figure S11: Comparison of newly defined baseline QSAR for neutral chemicals based on a critical membrane burden (CMB) of 26 mmol/L_{lip} for AREc32 and ARE-*bla* (A) and for GR-*bla* (B) with the QSAR using a CMB of 69 mmol/L_{lip} from Escher et al. (2019). 14

References. 14

Excel Table S1: Chemical information and physicochemical properties of the test chemicals. ID, DTXSID, molecular mass, CAS number, provider, product number, purity, logarithmic octanol-water partitioning constant (log K_{ow}), logarithmic liposome-water distribution ratio (log $D_{lip/w}$), pK_a, charge at pH 7.4.

Text S1: Measurement of logarithmic octanol-water partition ratios (log K_{ow}) ratios of the test chemicals.

Log K_{ow} was measured for six ionizable chemicals (PTX014-019) using a Sirius T3 automated titrator (Pion) equipped with a glass Ag/AgCl pH electrode, a UV dip probe, a temperature probe, and a stirring unit. Experiments were performed at 25 °C with an ionic strength of 0.15 M KCl under an argon atmosphere using a potentiometric titration in the presence of octanol as partition solvent. Partitioning of the neutral species into the octanol results in a shift of the pK_a of the chemical. The direction and magnitude of the shift is dependents on the pK_a type (acid or base) and the lipophilicity of the compound, so if the measured pK_a in the presence of octanol and the aqueous pK_a can be used to calculate the lipophilicity, meaning the log K_{ow} of the chemical.[1,2] Approximately 1 mg of pure chemical was weighed in a Sirius T3 test vial. Log K_{ow} measurement was performed with the pH-metric low logP (PTX014-017) or medium logP (PTX018 and 019) assay. Three subsequential titrations were performed for each sample starting at pH 2 and titrating up to pH 12. The octanol percentage was 50 % for the low logP and 23 % for the medium logP in the first titration, 61 % and 44 % during the second titration and 68 % and 60 % for the third titration. Log K_{ow} of the chemicals were determined from the mean value of the three replicates using the Sirius T3 Refine software (version 2.0.0.0.).

Table S2: Hydrophilic chemicals for baseline QSAR. CAS, provider, purity and experimental logarithmic liposome-water distribution ratio (log $D_{lip/w}$).

Chemical	CAS	Provider	Purity (%)	Experimental log $D_{lip/w}$[3]	pK_a[4]
1,2-trans-Cyclohexanediol	1460-57-7	Sigma-Aldrich	98.0	0.23	14.9
1,7-Heptanediol	629-30-1	Sigma-Aldrich	95.0	0.32	15.6
1,3-Cyclohexanedione	504-02-9	Sigma-Aldrich	97.0	0.49	5.4
2-Butoxyethanol	111-76-2	Sigma-Aldrich	99.5	0.60	14.9
5-Hexyn-1-ol	928-90-5	Sigma-Aldrich	96.0	0.78	15.9
1,2-Hexanediol	6920-22-5	Sigma-Aldrich	98.0	0.81	14.9
1,8-Octanediol	629-41-4	Sigma-Aldrich	98.0	0.81	15.6
Glycerol	56-81-5	Sigma-Aldrich	99.0	-1.04	14.2; 15.9
Ethylene glycol	107-21-1	Sigma-Aldrich	99.8	-0.79	14.8
1,5-Pentanediol	111-29-5	Sigma-Aldrich	97.0	-0.70	15.5
2,4-Pentanediol	625-69-4	Sigma-Aldrich	98.0	-0.30	15.4
Hexane-1,5-diol	928-40-5	Sigma-Aldrich	99.0	-0.22	15.6
Butanamide	541-35-5	Sigma-Aldrich	98.0	-0.20	-0.3; 15.1
1,6-Hexanediol	629-11-8	Sigma-Aldrich	99.0	-0.10	15.6

Table S3: Protein and lipid contents of the bioassay media used for the three assays taken from Qin et al. (2024).[5] FBS = fetal bovine serum, cs-FBS = charcoal-stripped FBS.

Assay	Medium for QSAR	$VF_{protein,medium}$ [L/L]	$VF_{lipid,medium}$ [L/L]	$VF_{water,medium}$ [L/L]	$V_{total,medium}$ [µL]
AREc32	10 % FBS generic	3.00×10^{-3}	7.00×10^{-5}	9.97×10^{-1}	40
ARE-*bla*					40
GR-*bla*	2% cs-FBS	9.40×10^{-4}	1.47×10^{-5}	9.99×10^{-1}	40

Text S2: Optimization of EROD, EFCOD and BFCOD assay.

Effect concentrations for the CYP induction of omeprazole and benzo[a]pyrene (BaP) were obtained from the literature (Table S4). Cytotoxicity was tested for all three cell lines before the test. Both chemicals were tested in serial dilution as described for the other test chemicals. All effect concentrations can be found in Table S4 and concentration response curves can be found in Figures S1-S3. Concentrations for 7-Ethoxyresorufin-O-deethylase (EROD), 7-Ethoxy-4-trifluoromethyl coumarin O-deethylase (EFCOD) and benzyloxy-4-trifluoromethylcoumarin O-debenzyloxylase (BFCOD) assays were kept below IC_{10} values. The test concentrations for both CYP inducers can be found in Table S4.

To find out the best substrate concentration for EROD, EFCOD and BFCOD assay, different concentrations of 7-ethoxyresorufin (ETX, 1.60×10^{-5} – 1.25×10^{-7} M), 7-ethoxy-4-

trifluoro-methylcoumarin (EFC, $4.00 \times 10^{-5} - 3.13 \times 10^{-7}$ M) and 7-benzyloxy-4-trifluoromethylcoumarin (BFC, $2.00 \times 10^{-5} - 7.80 \times 10^{-8}$ M) were tested after exposure of AREc32 cells with the inducers (omeprazole and BaP) (Figure S4). Concentrations for both inducers can be found in Table S4. For both inducers, the highest CYP activity was measured at an ETX concentration of approximately 2 µM, so this concentration was selected for the EROD assay. For the EFCOD and BFCOD assays no clear trend could be determined, so a concentration of 5 µM EFC or BFC was selected.

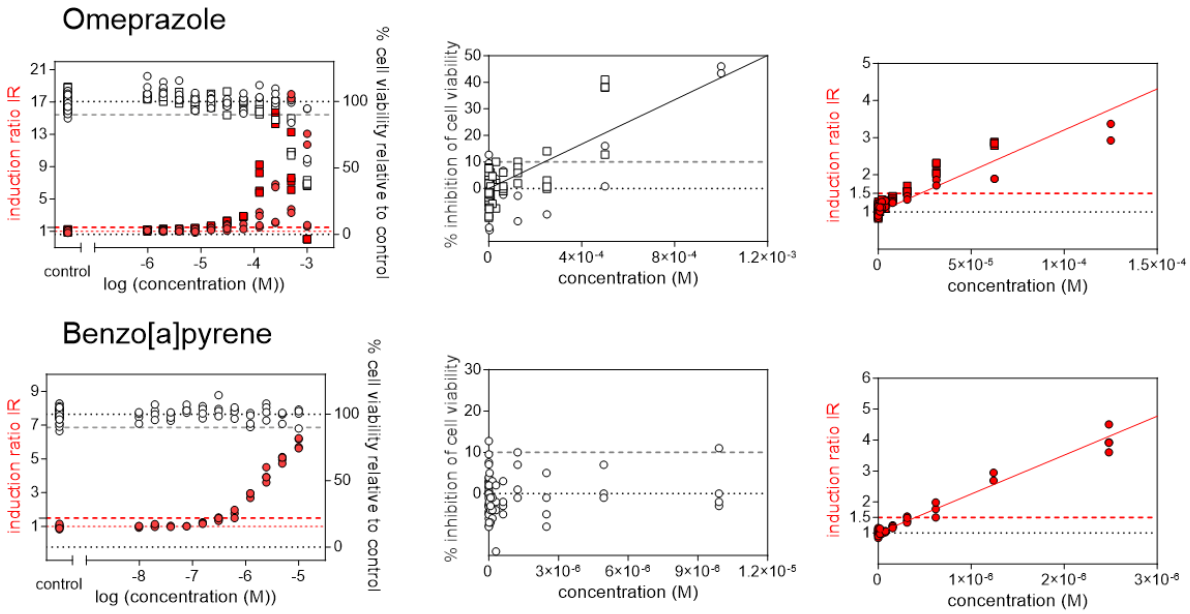

Figure S1: Concentration-response curves (CRC) of the CYP inducers in the AREc32 assay. Left: Full CRC; red: induction ratio and white: cell viability compared to control plotted against the logarithm of chemical concentration. Middle: Linear part of CRC; induction ratio plotted against chemical concentration. Right: Linear part of CRC; cell viability inhibition plotted against chemical concentration.

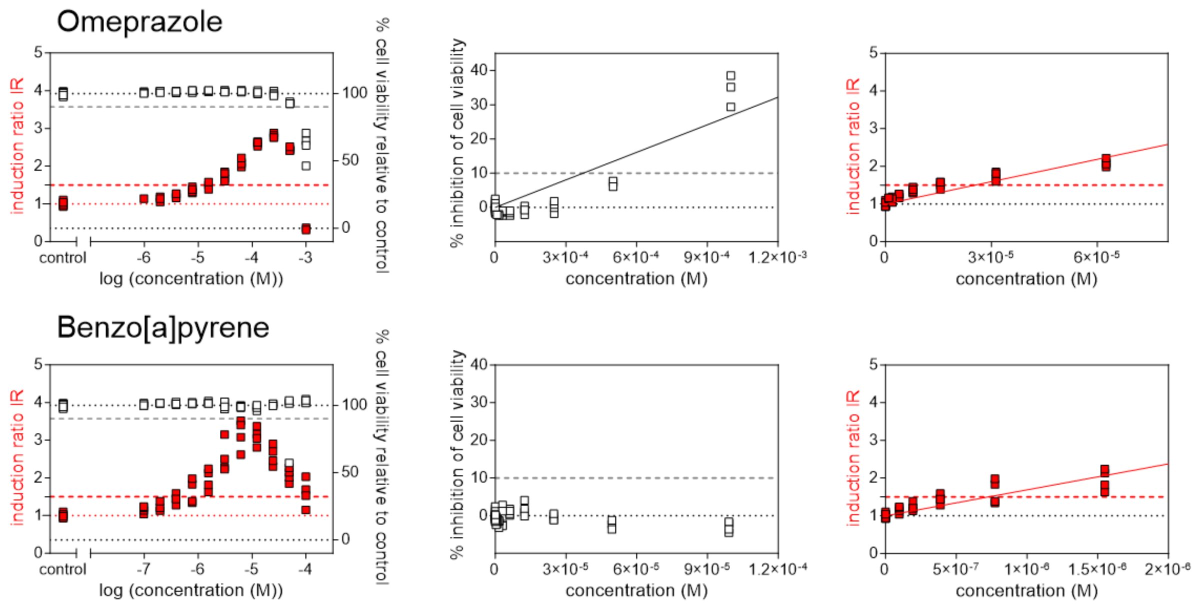

Figure S2: Concentration-response curves (CRC) of the CYP inducers in the ARE-*bla* assay. Left: Full CRC; red: induction ratio and white: cell viability compared to control plotted against the logarithm of chemical concentration. Middle: Linear part of CRC; induction ratio plotted against chemical concentration. Right: Linear part of CRC; cell viability inhibition plotted against chemical concentration.

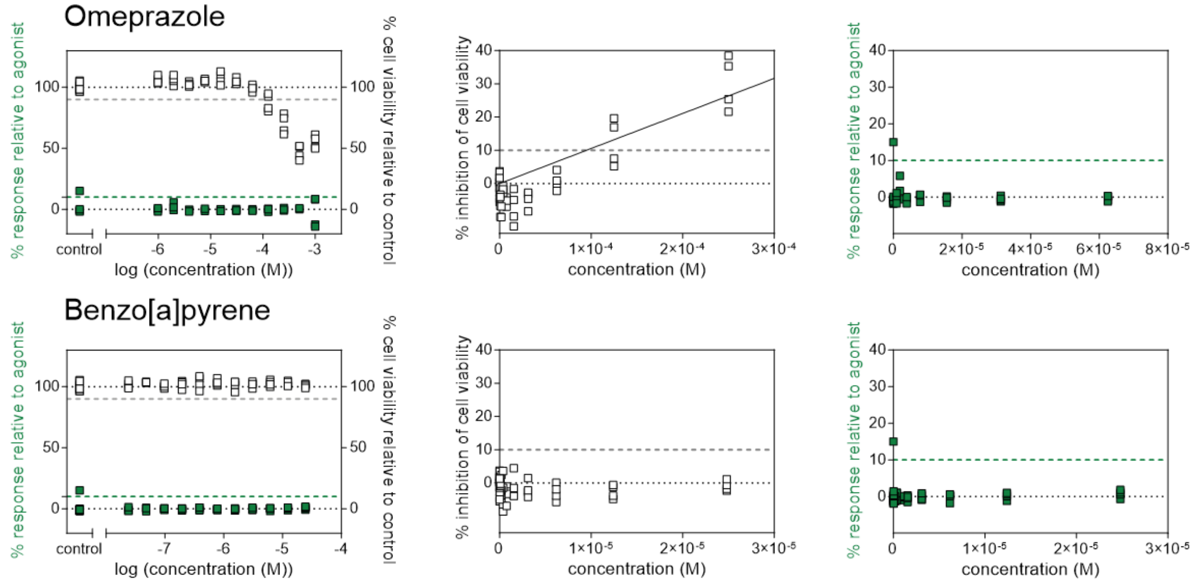

Figure S3: Concentration-response curves (CRC) of the CYP inducers in the GR-*bla* assay. Left: Full CRC; green: reporter gene activation relative to agonist and white: cell viability compared to control plotted against the logarithm of chemical concentration. Middle: Linear part of CRC; reporter gene activation relative to agonist plotted against chemical concentration. Right: Linear part of CRC; cell viability inhibition plotted against chemical concentration.

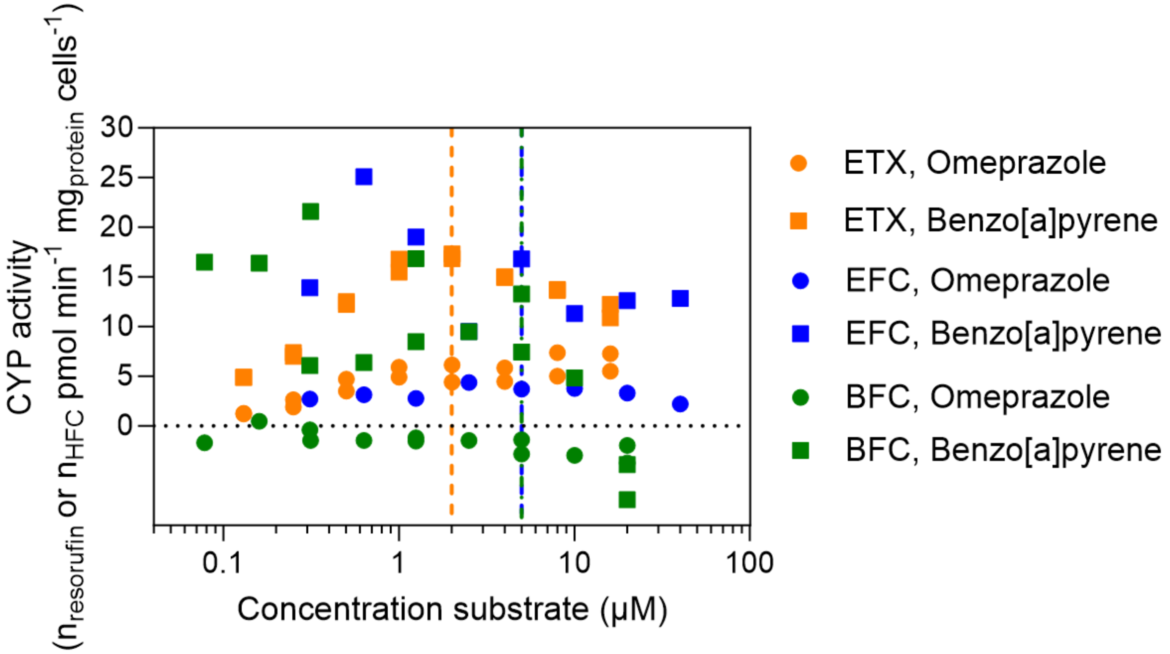

Figure S4: Optimization of 7-ethoxyresorufin (ETX), 7-ethoxy-4-trifluoromethylcoumarin (EFC) and 7-benzyloxy-4-trifluoromethylcoumarin (BFC) concentrations. CYP activity is displayed as amount of resorufin ($n_{resorufin}$) for EROD assay or amount of 7-hydroxy-4-trifluoromethylcoumarin (n_{HFC}) for EFCOD and BFCOD assay formed per minute and per $mg_{protein}$. The orange dashed line shows the final ETX concentration (2 µM). and the blue and green dashed line shows the final EFC and BFC concentration (5 µM).

Table S4: Effect concentrations of CYP inducers.

**Chemical**	**Omeprazole**		**Benzo[a]pyrene**	
AREc32 IC_{10} / $EC_{IR1.5}$ (M)	$2.39 \times 10^{-4} \pm 1.87 \times 10^{-5}$	$2.26 \times 10^{-5} \pm 8.65 \times 10^{-7}$	Not toxic up to 9.91×10^{-6}	$3.98 \times 10^{-7} \pm 7.43 \times 10^{-9}$
AREc32 concentration for CYP induction assay (M)	4.80×10^{-5} (EROD), 1.98×10^{-6} (EFCOD), 9.60×10^{-5} (BFCOD)		1.98×10^{-6} (EROD, BFCOD), 2.48×10^{-6} (EFCOD)	
ARE-*bla* IC_{10} / $EC_{IR1.5}$ (M)	$3.73 \times 10^{-4} \pm 2.42 \times 10^{-5}$	$2.53 \times 10^{-5} \pm 1.21 \times 10^{-6}$	Not active up to 9.91×10^{-5}	
ARE-*bla* concentration for CYP induction assay (M)	2.33×10^{-5}		9.90×10^{-6}	
GR-*bla* IC_{10} / EC10 (M)	$9.48 \times 10^{-5} \pm 9.53 \times 10^{-6}$	Not active up to cytotoxicity	Not active up to 2.48×10^{-5}	$5.40 \times 10^{-10} \pm 3.76 \times 10^{-11}$
GR-*bla* concentration for CYP induction assay (M)	5.93×10^{-5}		1.55×10^{-6}	
Effect concentration CYP induction (M)	1.00×10^{-4} [[6]]		9.91×10^{-7} [[7]]	

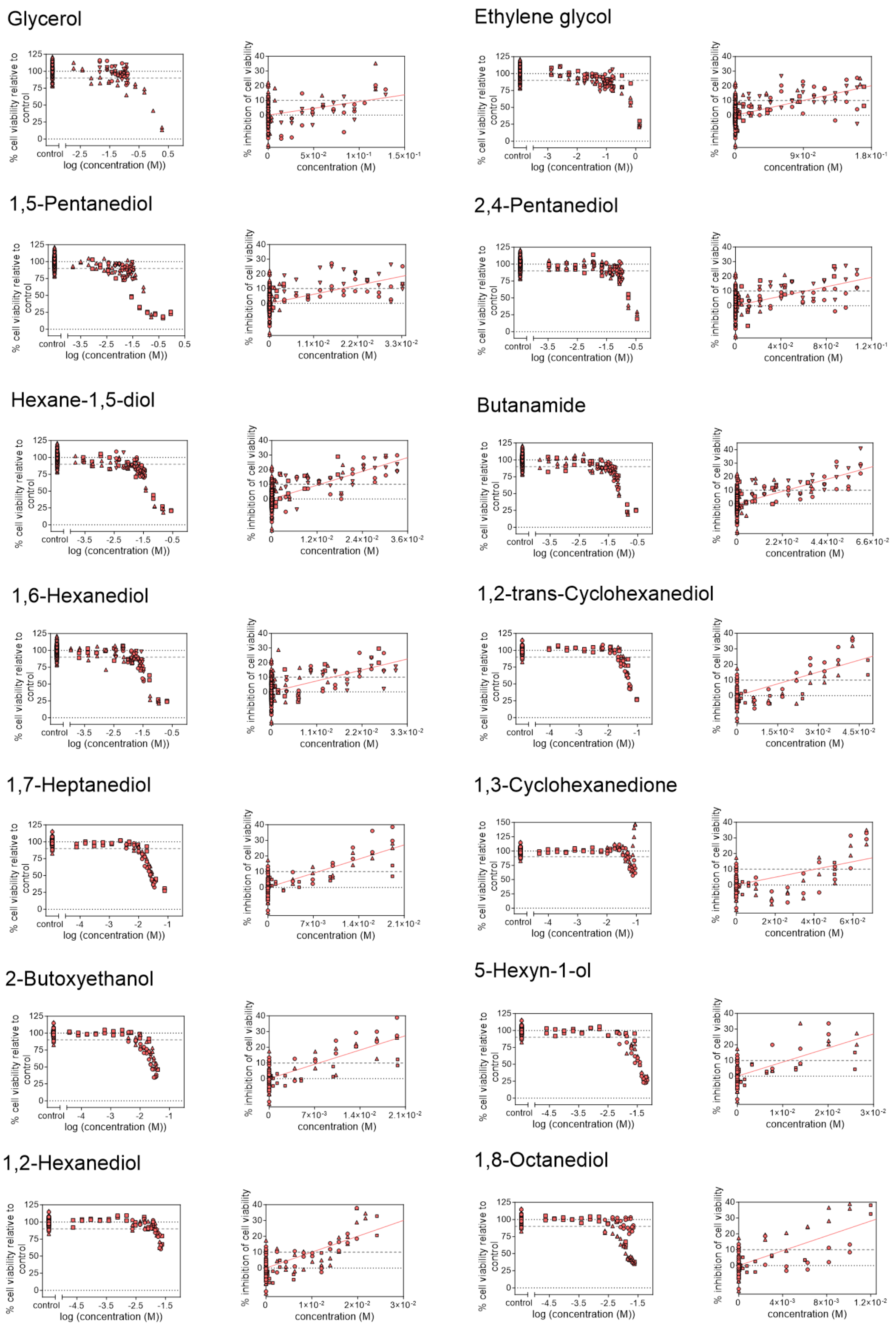

Figure S5: Concentration response curves (CRC) for hydrophilic chemicals in the AREc32 assay. The left graph shows the full CRC of cell viability relative to control plotted against logarithmic concentration. The right graph shows the linear part of the CRC for inhibition of cell viability plotted against concentration.

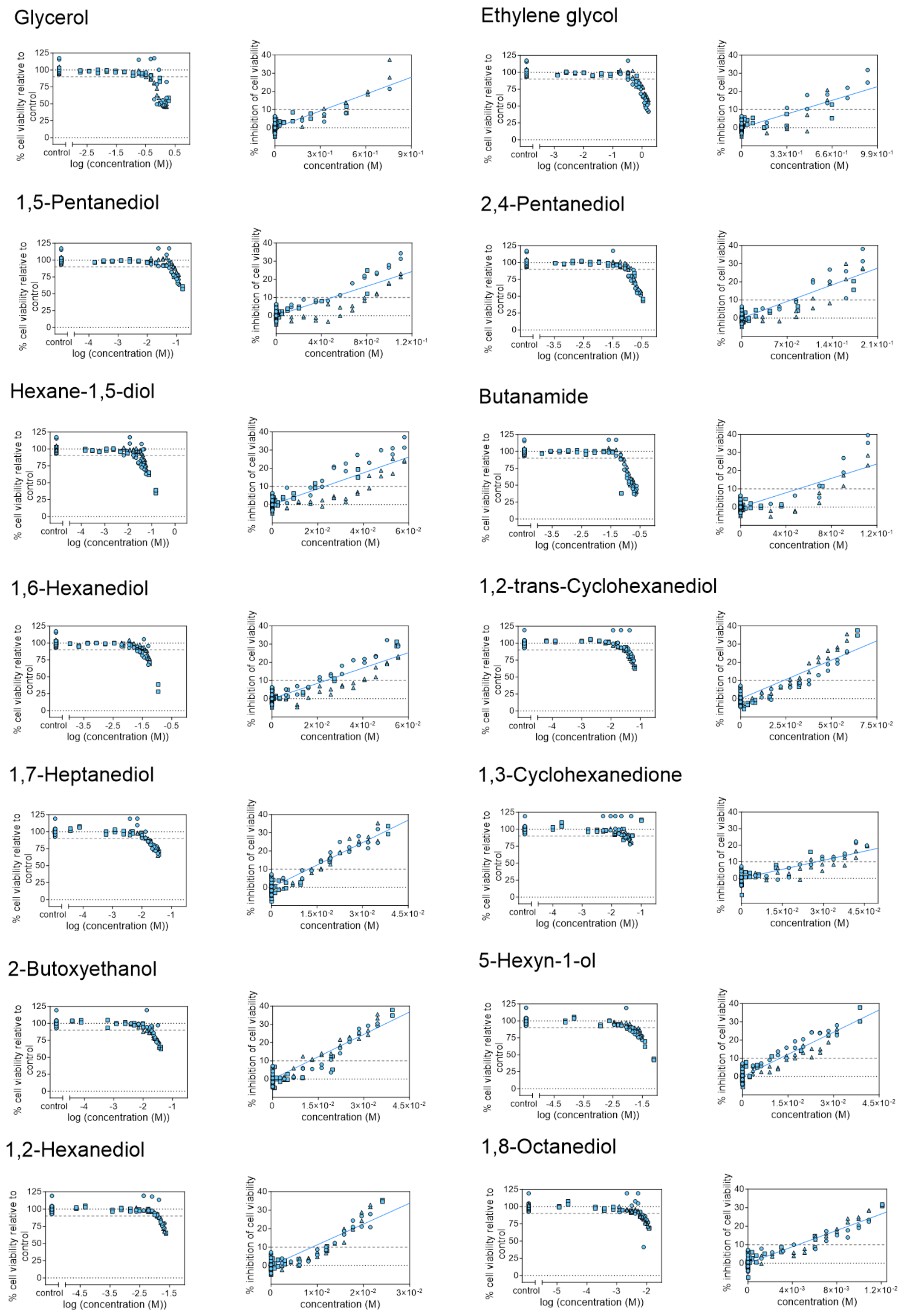

Figure S6: Concentration response curves (CRC) for hydrophilic chemicals in the ARE-*bla* assay. The left graph shows the full CRC of cell viability relative to control plotted against logarithmic concentration. The right graph shows the linear part of the CRC for inhibition of cell viability plotted against concentration.

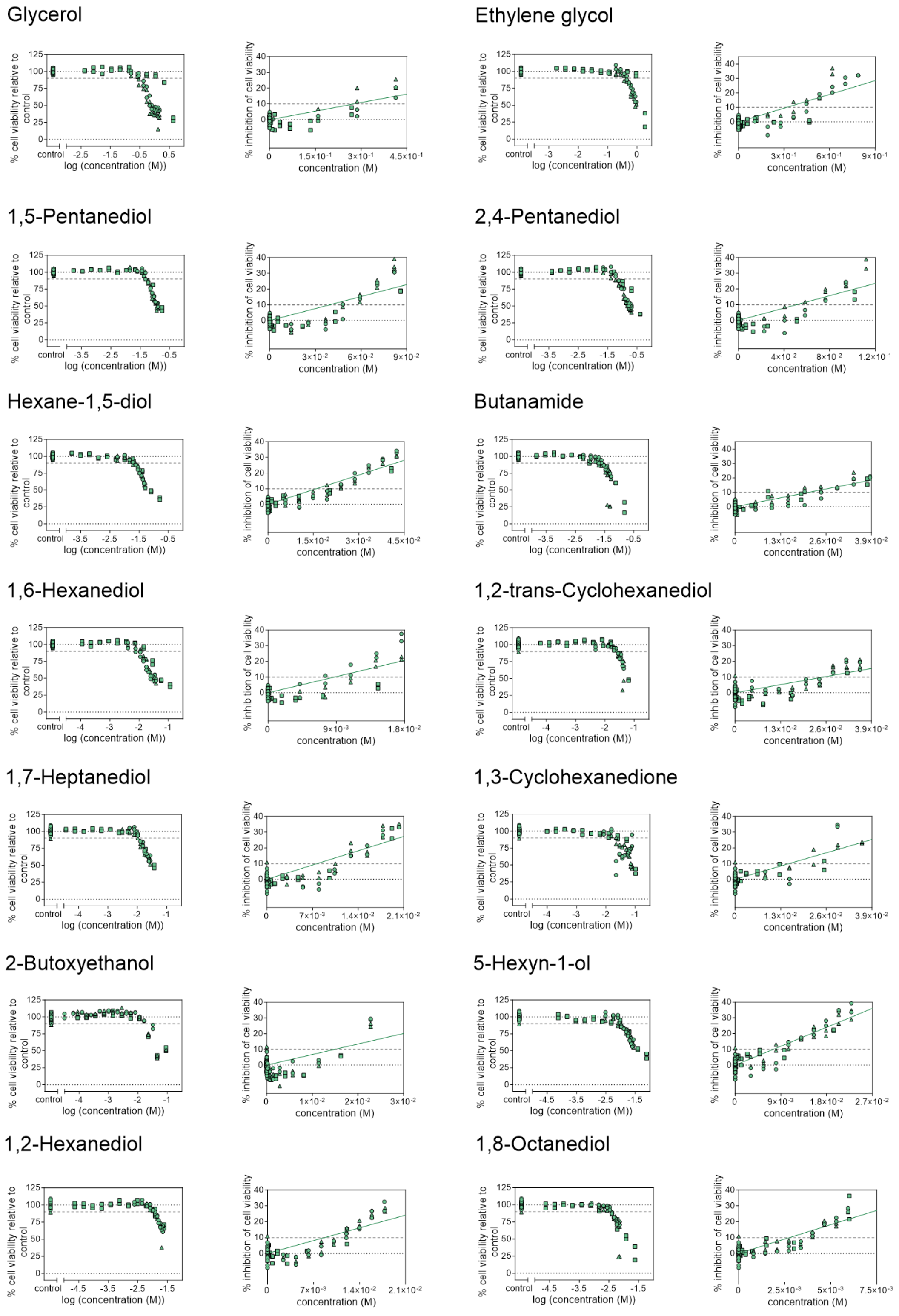

Figure S7: Concentration response curves (CRC) for hydrophilic chemicals in the GR-*bla* assay. The left graph shows the full CRC of cell viability relative to control plotted against logarithmic concentration. The right graph shows the linear part of the CRC for inhibition of cell viability plotted against concentration.

Table S5: Concentration causing a reduction of cell viability of 10 % (IC_{10}) for hydrophilic chemicals.

Chemical	IC_{10} AREc32 [M]	CV [%]	IC_{10} ARE-*bla* [M]	CV [%]	IC_{10} GR-*bla* [M]	CV [%]
Glycerol	1.09×10^{-1}	22.1	3.24×10^{-1}	5.3	2.76×10^{-1}	11.7
Ethylene glycol	8.95×10^{-2}	7.9	4.37×10^{-1}	7.1	3.16×10^{-1}	7.0
1,5-Pentanediol	1.80×10^{-2}	9.1	4.93×10^{-2}	5.3	3.94×10^{-2}	8.3
2,4-Pentanediol	6.20×10^{-2}	8.6	7.63×10^{-2}	5.1	5.09×10^{-2}	8.3
Hexane-1,5-diol	1.27×10^{-2}	5.9	2.29×10^{-2}	5.1	1.60×10^{-2}	3.5
Butanamide	2.40×10^{-2}	6.1	5.06×10^{-2}	8.0	2.11×10^{-2}	4.7
1,6-Hexanediol	1.47×10^{-2}	8.3	2.37×10^{-2}	4.1	8.73×10^{-3}	9.4
1,2-trans-Cyclohexanediol	1.97×10^{-2}	7.3	2.36×10^{-2}	3.9	2.51×10^{-2}	7.7
1,7-Heptanediol	7.75×10^{-3}	5.4	1.22×10^{-2}	2.4	7.70×10^{-3}	5.7
1,3-Cyclohexanedione	4.04×10^{-2}	11.5	2.75×10^{-2}	4.9	1.54×10^{-2}	7.5
2-Butoxyethanol	7.67×10^{-3}	5.7	1.22×10^{-2}	3.0	1.49×10^{-2}	15.1
5-Hexyn-1-ol	1.11×10^{-2}	8.2	1.23×10^{-2}	2.8	7.50×10^{-3}	3.6
1,2-Hexanediol	9.97×10^{-3}	6.5	8.88×10^{-3}	3.6	8.70×10^{-3}	6.5
1,8-Octanediol	4.26×10^{-3}	8.5	4.52×10^{-3}	2.7	2.77×10^{-3}	4.7

Table S6: Freely dissolved concentration causing a reduction of cell viability of 10 % ($IC_{10,free}$) and logarithmic liposome-water distribution ratios for AREc32 assay for 14 ionizable organic chemicals and caffeine from Huchthausen et al. (2020).[8]

Chemical	$IC_{10,free}$ AREc32 [M]	CV [%]	Log $D_{lip/w}$ [L/L]
Diclofenac	3.12×10^{-5}	21.6	2.64
2,4-Dichlorophenoxyacetic acid (2,4D)	1.32×10^{-4}	21.9	2.02
Naproxen	3.06×10^{-4}	28.3	2.17
Torasemide	1.84×10^{-4}	27.1	2.05
Warfarin	9.56×10^{-5}	8.0	1.62
Ibuprofen	7.09×10^{-5}	6.3	1.81
Venlafaxin	4.51×10^{-4}	4.7	1.64
Metoprolol	7.89×10^{-4}	13.8	1.42
Diphenhydramine	1.69×10^{-4}	6.0	2.17
Propranolol	1.47×10^{-4}	11.3	2.73
Lamotrigine	5.00×10^{-4}	9.0	2.06
Telmisartan	2.62×10^{-6}	5.2	4.73
Genistein	2.96×10^{-5}	7.9	3.32
Labetalol	8.90×10^{-5}	7.2	3.26
Caffeine	4.33×10^{-3}	7.7	0.08

Excel Table S7: *In vitro* effect concentrations of all chemicals in AREc32, ARE-*bla* and GR-*bla* assay. $IC_{10,baseline}$, highest tested concentration, IC_{10}, $IC_{10,ToxBLAzer}$, $EC_{IR1.5}$, EC_{10}, standard errors (SE), toxic ratio (TR), specificity ratios ($SR_{cytotoxicity}$ and $SR_{baseline}$). IC_{10} and $EC_{IR1.5}$ for AA and NMBAA-MAA are taken from Huchthausen et al. (2023).

Excel Table S8: Concentration-response curves (CRC) of all chemicals in all assays for cytotoxicity, ToxBLAzer cytotoxicity, oxidative stress response activation and glucocorticoid receptor (GR) activation. Data for AA and NMBAA - MAA was taken from Huchthausen et al. (2023).

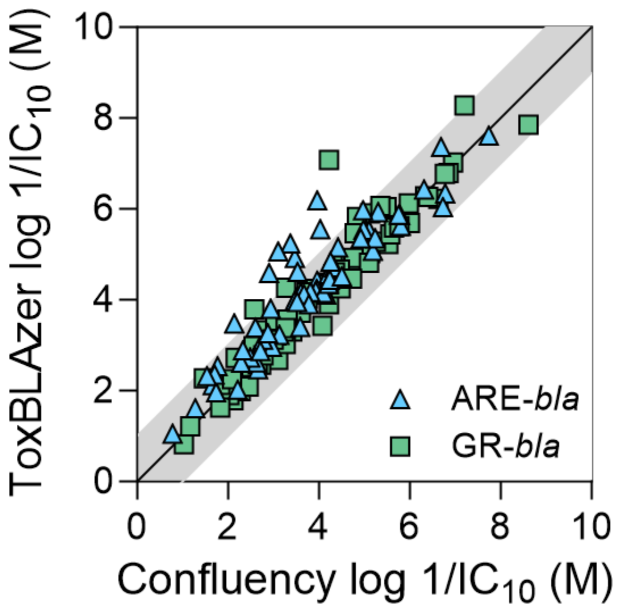

Figure S8: Comparison of cytotoxicity determined with confluency measurement (Confluency log $1/IC_{10}$) or with the ToxBLAzer™ reagent (ToxBLAzer log $1/IC_{10}$). Blue triangles represent the results for ARE-*bla* and green squares represent results for GR-*bla*. No data for AREc32 is shown since the cell line uses luciferase as reporter enzyme instead of beta-lactamase. The black line indicates a perfect agreement of both methods and the grey areas indicate a deviation of a factor of ten.

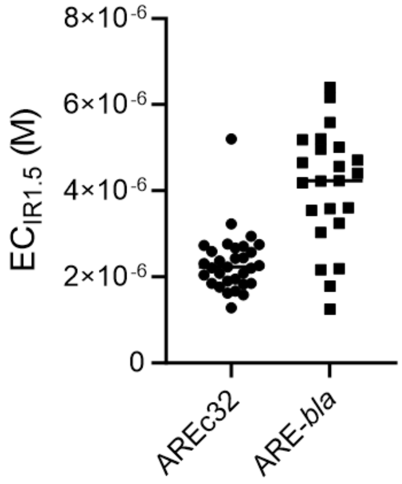

Figure S9: Oxidative stress response activation ($EC_{IR1.5}$) of reference compound *tert*-butylhydroquinone (tBHQ) for AREc32 and ARE-*bla* assay. The data points show results for the different plates.

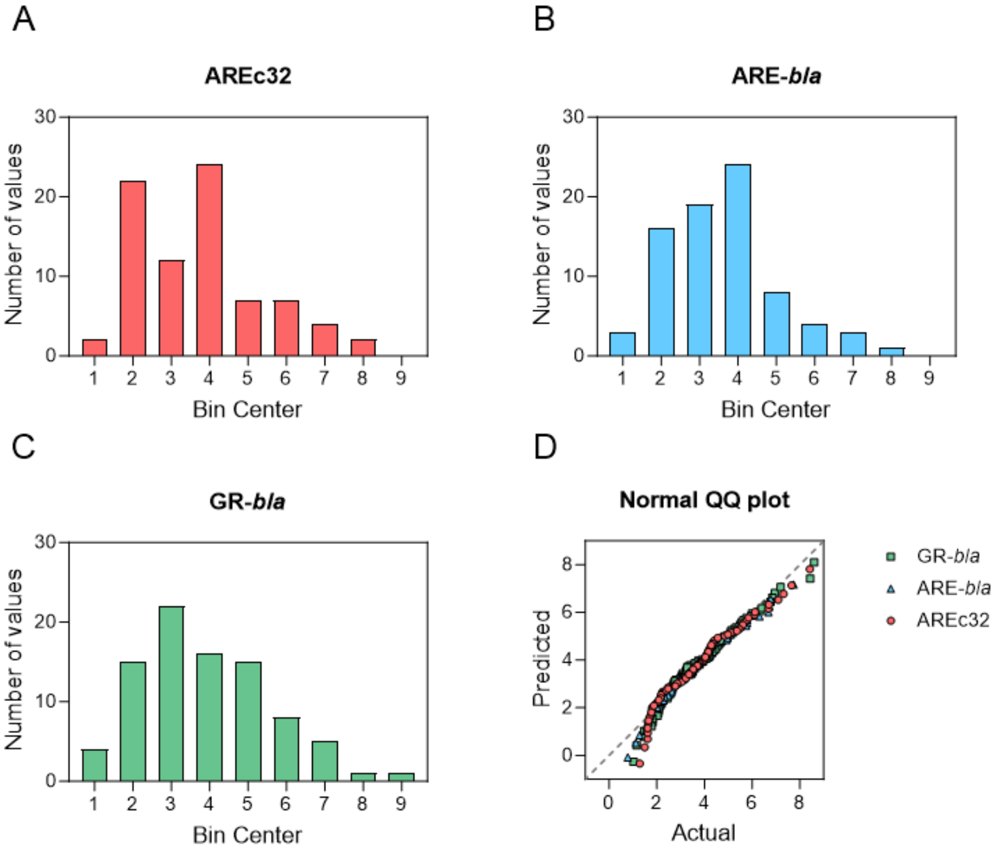

Figure S10: Normal distribution of bioassay cytotoxicity data (log $1/IC_{10}$). A-C show density plots of AREc32, ARE-*bla* and GR-*bla.* D shows QQ plot of all data sets.

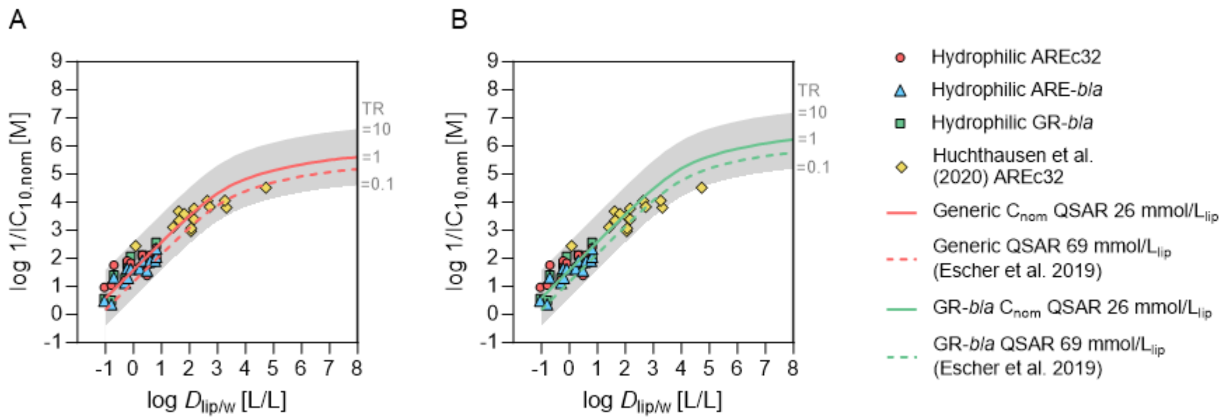

Figure S11: Comparison of newly defined baseline QSAR for neutral chemicals based on a critical membrane burden (CMB) of 26 mmol/L_{lip} for AREc32 and ARE-*bla* (A) and for GR-*bla* (B) with the QSAR using a CMB of 69 mmol/L_{lip} from Escher et al. (2019).[9]

References.

1. Avdeef A, Box KJ, Takacs-Novak K. pH-metric log P. 6. Effects of sodium, potassium, and N-CH3-D-glucamine on the octanol-water partitioning of prostaglandins E1 and E2. *J Pharm Sci.* 1995;84(5):523-529.
2. Avdeef A. pH-metric log P. II: Refinement of partition coefficients and ionization constants of multiprotic substances. *J Pharm Sci.* 1993;82(2):183-190.
3. Endo S, Escher BI, Goss K-U. Capacities of Membrane Lipids to Accumulate Neutral Organic Chemicals. *Environ Sci Technol.* 2011;45(14):5912-5921.
4. ACD/Labs. ACD/ Percepta. 1994-2015; Build 2726.
5. Qin W, Henneberger L, Glüge J, König M, Escher BI. Baseline toxicity model to identify the specific and non-specific effects of per- and polyfluoroalkyl substances in cell-based bioassays *Environ Sci Technol.* 2024;58(13):5727–5738.
6. Choi JM, Oh SJ, Lee SY, Im JH, Oh JM, Ryu CS, Kwak HC, Lee JY, Kang KW, Kim SK. HepG2 cells as an in vitro model for evaluation of cytochrome P450 induction by xenobiotics. *Arch Pharm Res.* 2015;38(5):691-704.
7. Fischer FC, Abele C, Henneberger L, Klüver N, König M, Mühlenbrink M, Schlichting R, Escher BI. Cellular Metabolism in High-Throughput In Vitro Reporter Gene Assays and Implications for the Quantitative In Vitro-In Vivo Extrapolation. *Chem Res Toxicol.* 2020;33(7):1770-1779.
8. Huchthausen J, Mühlenbrink M, König M, Escher BI, Henneberger L. Experimental Exposure Assessment of Ionizable Organic Chemicals in In Vitro Cell-Based Bioassays. *Chem Res Toxicol.* 2020;33(7):1845-1854.
9. Escher BI, Glauch L, König M, Mayer P, Schlichting R. Baseline Toxicity and Volatility Cutoff in Reporter Gene Assays Used for High-Throughput Screening. *Chem Res Toxicol.* 2019;32(8):1646-1655.
